# Enhancing photoelectrochemical water splitting by combining work function tuning and heterojunction engineering

**DOI:** 10.1038/s41467-019-11586-y

**Published:** 2019-08-15

**Authors:** Kai-Hang Ye, Haibo Li, Duan Huang, Shuang Xiao, Weitao Qiu, Mingyang Li, Yuwen Hu, Wenjie Mai, Hongbing Ji, Shihe Yang

**Affiliations:** 1 Fine Chemical Industry Research Institute, School of Chemistry, Sun Yat-sen University, 510275 Guangzhou, China; 2 Guangdong Key Lab of Nano-Micro Material Research, School of Chemical Biology and Biotechnology, Shenzhen Graduate School, Peking University, Xili University Town, 518055 Shenzhen, China; 3 Siyuan Laboratory, Guangzhou Key Laboratory of Vacuum Coating Technologies and New Energy Materials, Guangdong Provincial Engineering Technology Research Center of Vacuum Coating Technologies and New Energy Materials, Department of Physics, Jinan University, 510632 Guangzhou, China; 4 Department of Chemistry, The Hong Kong University of Science and Technology, 999077 Clear Water Bay, Kowloon, Hong Kong China

**Keywords:** Electronic materials, Devices for energy harvesting, Photocatalysis

## Abstract

We herein demonstrate the unusual effectiveness of two strategies in combination to enhance photoelectrochemical water splitting. First, the work function adjustment via molybdenum (Mo) doping significantly reduces the interfacial energy loss and increases the open-circuit photovoltage of bismuth vanadate (BiVO_4_) photoelectrochemical cells. Second, the creation and optimization of the heterojunction of boron (B) doping carbon nitride (C_3_N_4_) and Mo doping BiVO_4_ to enforce directional charge transfer, accomplished by work function adjustment via B doping for C_3_N_4_, substantially boost the charge separation of photo-generated electron-hole pairs at the B-C_3_N_4_ and Mo-BiVO_4_ interface. The synergy between the above efforts have significantly reduced the onset potential, and enhanced charge separation and optical properties of the BiVO_4_-based photoanode, culminating in achieving a record applied bias photon-to-current efficiency of 2.67% at 0.54 V vs. the reversible hydrogen electrode. This work sheds light on designing and fabricating the semiconductor structures for the next-generation photoelectrodes.

## Abstract (web summary)

While photoelectrodes represent a promising solar-to-fuel conversion technology, material challenges limit performances. Here, authors improve the onset potential and charge separation of bismuth vanadate photoanode water splitting performances by work function tuning and heterojunction engineering.

## Introduction

Photoelectrochemical cell (PEC) for water splitting is a key technology of the future for hydrogen production^1–3^. Despite the widespread attention that has been received, this technology still has many hurdles to overcome and uncharted territories to explore. Ultimately, the photon to hydrogen conversion efficiency has yet to be increased to such a level that commercial applications could become viable^4^.

In the PEC water splitting process, photons are first absorbed by the photoelectrode producing electrons and holes, which are then separated and participated in the hydrogen evolution reaction (HER) on cathode and the oxygen evolution reaction (OER) on anode, possibly with the assistance of a bias voltage^1^. Therefore, one way to enhance the PEC efficiency is to increase the quantum efficiency of photons in a PEC system by improving the efficiencies of light harvesting, charge separation and surface charge transfer^3–10^. Another strategy to enhance the PEC efficiency is to minimize the overpotential by reducing the voltage loss related to charge recombination, sluggish surface kinetics, etc.

BiVO_4_ has received great attention in recent years because it is a promising sustainability-inspired photoanode material for PEC with a suitable band gap for visible light absorption^11^ and favorable conduction band edge position (0.1–0.2 V vs. NHE) for H_2_ evolution^7^. However, the PEC efficiency at low bias voltages of BiVO_4_ photoanode still has much room for improvement^11–13^. In particular, due to the presence of numerous trap states and surface defects as well as the associated surface Fermi-level pinning effect, the BiVO_4_ films variously prepared so far are still plagued by the quite low open-circuit photo-voltage when used as photoanodes^11,14,15^. To address this issue, doped photoanodes, such as W-BiVO_4_, Mo-BiVO_4_, have been fabricated aiming to enhance charge transport and to reduce the charge recombination^11,12,16,17^. Meanwhile, W-BiVO_4_/BiVO_4_, Co_2_O_3_/BiVO_4_, and BiOI/BiVO_4_ photoanodes have been developed in the form of so-called homojunctions and heterojunctions to enhance the charge separation in PEC systems^6,13,18^. Other problems of the BiVO_4_-based photoanode include the still low coverage of the solar spectrum which it is able to harvest as well as the low charge separation efficiency. To address these problems, carbon quantum dots/BiVO_4_ and nitrogen doped BiVO_4_ photoanodes have been reported showing broadened light absorption range, enhanced light harvesting efficiency, and boosted interfacial charge transfer for PEC water splitting^9,18^. As for improving the utilization efficiency of surface charge for oxygen evolution, the combined catalyst/photoelectrode systems, such as FeOOH/BiVO_4_, NiFeO_x_/BiVO_4_, Co-Pi/BiVO_4_, and NiOOH/FeOOH/BiVO_4_, have been commonly used^7,19–21^.

Recent efforts have improved the photocurrent density of BiVO_4_ based photoanodes for water splitting to nearly 90% of its theoretical value at 1.23 V vs. RHE^22,23^. However, the photon to hydrogen conversion efficiency is still far from its theoretical value mainly due to the stagnant carrier transport. Especially when a PEC cell works at low bias, the carrier transport is more susceptible to blockage by any potential barriers in the energy landscape along the carrier passage. Specifically, poor performance at low bias of BiVO_4_ based photoanodes led to poor applied bias photon-to-current efficiencies (ABPEs) as reported in some PEC water splitting systems, such as Bi-NiFeO_x_/BiVO_4_ (2.25%)^20^, NiOOH/FeOOH/BiVO_4_ (1.75%)^7^, NiO/CoO_x_/BiVO_4_ (1.5%)^11^, NiOOH/FeOOH/N-BiVO_4_ (2.2%)^9^. Previously, nanostructures and cocatalysts have been used to promote photocurrents and to minimize onset potentials, respectively^7,19–21^.

Meanwhile, because C_3_N_4_ has a favorable conduction band edge position relative to that of BiVO_4_, a heterojunction between the two could increase the charge separation. Prompted by this expectation, the conjugation of C_3_N_4_ with BiVO_4_ has received great attention in recent years^24–28^. However, before the C_3_N_4_/BiVO_4_ junction could efficiently drive the PEC water splitting, new strategies must be developed to elaborate the band structure at the junction to optimize charge separation by minimizing interfacial kinetic barriers and energy losses.

In this work, we endeavored to explore such ways to further improve the PEC performance of the BiVO_4_ photoanode. First, we systematically studied the effect of Mo doping on the electron band structure of BiVO_4_, and discovered that a moderate Mo doping of BiVO_4_, a low end doping regime that has not been explored before, can increase the photo-voltage of BiVO_4_ photoanodes from 0.24 V to ~1 V in 10 s irradiation. Second, to further improve the charge separation efficiency at low bias, we elaborated a cliff like junction between B-C_3_N_4_ and Mo-BiVO_4_, for which the band structure of C_3_N_4_ was judiciously tuned as well by B doping. With such an elaborated junction, interfacial charge transfer was remarkably enhanced. As the main thread running through this work, we make special efforts to advance our ability to modulate the work functions with a view to toning up the NiFeO_x_/B-C_3_N_4_/Mo-BiVO_4_ photoanodes for PEC water splitting. We have significantly increased the light harvesting efficiency (LHE) of the B-C_3_N_4_/Mo-BiVO_4_ photoanode, achieving photocurrent densities of 4.7 mA cm^−2^ at 0.6 V vs. RHE (*Φ*_Sep_ = 79%) and 6 mA cm^−2^ at 1.23 V vs. RHE (*Φ*_Sep_ = 98%) in potassium phosphate buffer (PPB) solution with 0.5 M Na_2_SO_3_ hole scavenger (pH 7). When the NiFeO_x_ was anchored on the B-C_3_N_4_/Mo-BiVO_4_ photoanode as an OER catalyst layer forming the NiFeO_x_/B-C_3_N_4_/Mo-BiVO_4_ photoanode, we obtained photocurrent densities of 3.85 mA cm^−2^ at 0.54 V vs. RHE (71% IPCE) and 5.93 mA cm^−2^ at 1.23 V vs. RHE (92% IPCE) in PPB solution without any hole scavengers such as Na_2_SO_3_ (pH 7). Significantly, the NiFeO_x_/B-C_3_N_4_/Mo-BiVO_4_ photoanode has achieved an ABPE up to 2.67% at 0.54 V vs. RHE, which is the highest reported to date and yet, with the lowest biased-voltage, for BiVO_4_-based PEC devices.

## Results

### Characterization of the NiFeO_x_/B-C_3_N_4_/Mo-BiVO_4_ photoanode

**Fig. 1:**
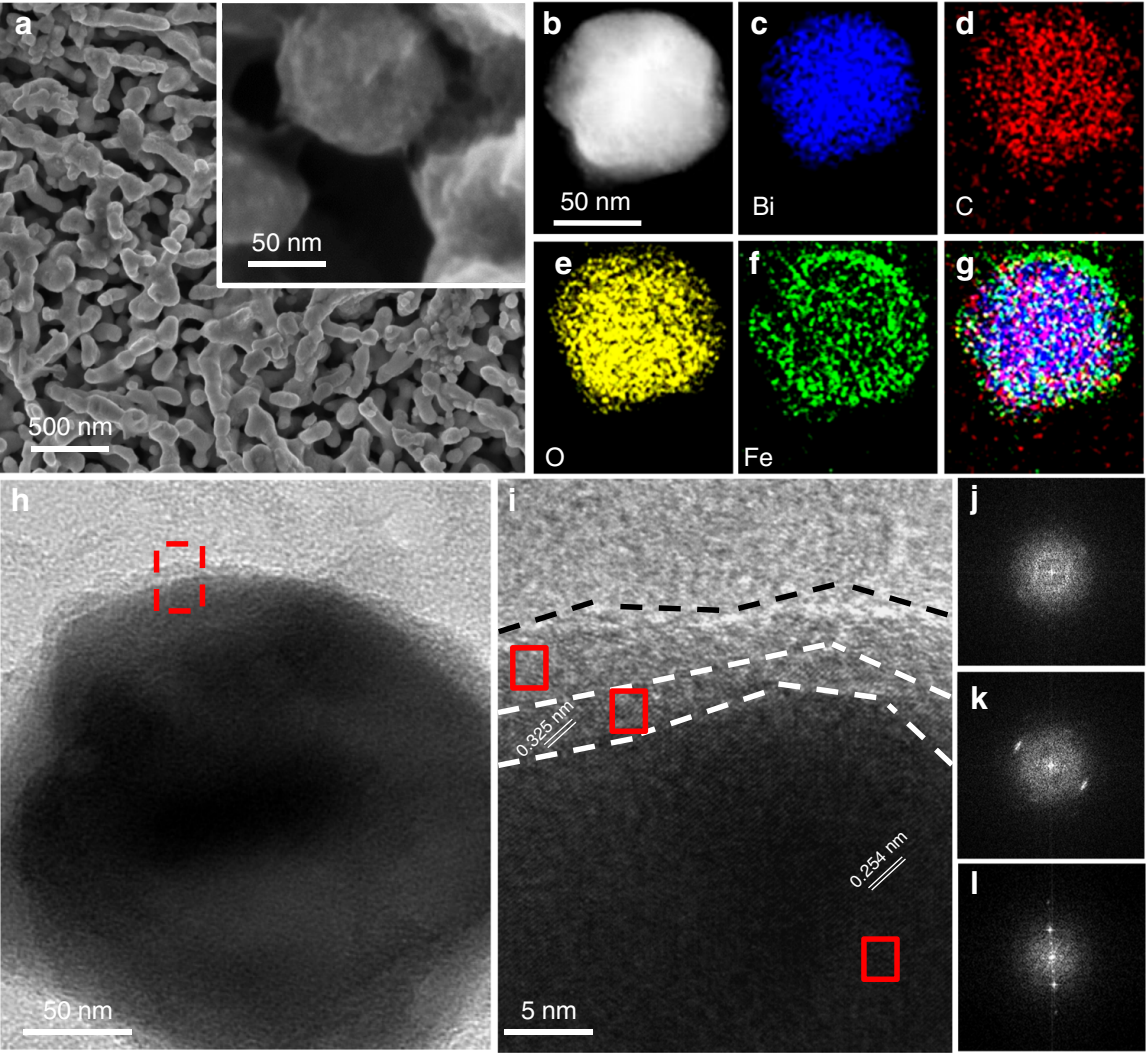
Electron microscopic characterization of the NiFeO_x_/B-C_3_N_4_/Mo-BiVO_4_ photoanodes. **a** Top-view (inset is the magnified image) SEM images of the NiFeO_x_/B-C_3_N_4_/Mo-BiVO_4_ photoanode. **b** HADDF-STEM image of the NiFeO_x_/B-C_3_N_4_/Mo-BiVO_4_ photoanode and **c**–**f** the corresponding STEM-EDS elemental mapping images for Bi, C, O, and Fe, respectively. **g** Overlay of the elemental mapping images of Fe, C, and Bi. **h** TEM, **i** HRTEM image of the NiFeO_x_/B-C_3_N_4_/Mo-BiVO_4_ photoanode and **j**–**l** corresponding diffraction patterns via fast Fourier transform of NiFeO_x_, B-C_3_N_4_, and Mo-BiVO_4_, respectively

The XRD patterns collected from BiVO_4_, Mo-BiVO_4_, B-C_3_N_4_/Mo-BiVO_4_, NiFeO_x_/B-C_3_N_4_/Mo-BiVO_4_ prepared on the F-doped SnO_2_ conducting glass (FTO), C_3_N_4_ and B-C_3_N_4_ are shown in Supplementary Fig. 1. All of the diffraction peaks in the XRD patterns obtained can be well indexed to monoclinic BiVO_4_ (JCPDS PDF #75–1866), SnO_2_ (JCPDS PDF #41–1445) and graphite-C_3_N_4_ (JCPDS PDF #50–1250)^18,29,30^. As shown in Fig. 1a, the champion photoanode consisted of nanoporous B-C_3_N_4_/Mo-BiVO_4_ heterojunctions in tandem with a NiFeO_x_ oxygen evolution catalyst (OEC) layer, which were successfully grown on the FTO substrate. (Supplementary Fig. 2) As shown in Fig. 1b–g, signals of Bi, C, O, and Fe elements are clearly observed in a row, suggesting that the newly-coated B-C_3_N_4_ has covered the nanoporous Mo-BiVO_4_, and NiFeO_x_ layer has covered the B-C_3_N_4_/Mo-BiVO_4_ as the OEC. Figures 1h, i is a typical TEM image and a high-resolution TEM (HRTEM) image of the NiFeO_x_/B-C_3_N_4_/Mo-BiVO_4_ sample, respectively, revealing that the B-C_3_N_4_ and Mo-BiVO_4_ are crystallized (Fig. 1j, k) while NiFeO_x_ is non-crystallized (Fig. 1l). The lattice fringes of 0.325 nm and 0.254 nm are ascribed to the (002) plane^31^ of C_3_N_4_ and the (020) plane^32^ of BiVO_4_, respectively.

### Work function tuning

**Fig. 2:**
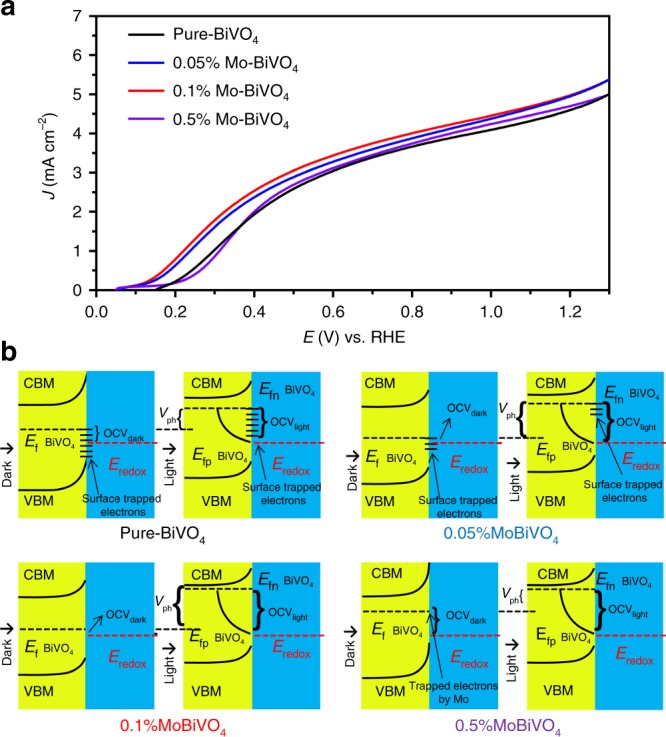
Photoelectrochemical and photo-voltage characterization of the BiVO_4_-based photoanodes. **a** LSV curves of pure-BiVO_4_, 0.05% Mo-BiVO_4_, 0.1% Mo-BiVO_4_, and 0.5% Mo-BiVO_4_ recorded at a scan rate of 25 mV s^−1^ under AM 1.5 G one-sun irradiation in PPB solution with 0.5 M Na_2_SO_3_ as a hole scavenger (pH 7). **b** Band structures and band bending schematics of pure-BiVO_4_, 0.05% Mo-BiVO_4_, 0.1% Mo-BiVO_4_, and 0.5% Mo-BiVO_4_, constructed from the XPS, UPS and photoelectrochemical measurement data

The first strategy we used to optimize the PEC performance of BiVO_4_-based photoanodes, more precisely, to lower the onset potential, was to systematically adjust the work function by Mo doping below 1%, which is a previously uncharted doping regime (the atomic ratio is shown in Supplementary Table 1). Figure 2a compares the linear sweep voltammograms (LSV) curves of BiVO_4_ (black line), 0.05% Mo doped BiVO_4_ (blue line) and 0.1% Mo doped BiVO_4_ (red line) 0.5% Mo doped BiVO_4_ (purple line) at a scan rate of 25 mV s^−1^ in 0.5 M Na_2_SO_3_ aqueous solution as a hole scavenger with PPB (pH 7) under AM 1.5 G one-sun illumination. The pure BiVO_4_ photoanode achieved photocurrent density of 3.2 (±0.3) mA cm^−2^ at 0.6 V vs. RHE and 4.7 (±0.3) mA cm^−2^ at 1.23 V vs. RHE, in agreement with the previous report^7^. With a slight doping, the photocurrent density of 0.1% Mo-BiVO_4_ increased to 3.4 (±0.2) mA cm^−2^ at 0.6 V vs. RHE and 4.98 (±0.2) mA cm^−2^ at 1.23 V vs. RHE. From our extensive measurements, the photocurrent densities assuming 100% absorbed photon-to-current efficiency (*J*_abs_) of the BiVO_4_-based and the Mo-BiVO_4_-based photoanodes were consistently ~4.7 mA cm^−2^ and ~5.01 mA cm^−2^, respectively (Supplementary Fig. 3). The increase in *J*_abs_ could be ascribed to the enhanced LHE, carrier concentration (Supplementary Fig. 27) and mobility (Supplementary Fig. 28) resulting from the Mo doping^33–37^. More interestingly, the onset potential of Mo doped BiVO_4_ (0.05, 0.1, and 0.5%) photoanodes became significantly more negative than that of pure BiVO_4_ photoanode, and the rapid photocurrent increase region against bias for the 0.05% and 0.1% Mo doped BiVO_4_ is also more negative than that of the BiVO_4_ photoanode. However, the rapid photocurrent increase region against bias for the 0.5% Mo doped BiVO_4_ is more positive than that of BiVO_4_. This phenomenon is caused by the changed open-circuit photo-voltage (OCP) of BiVO_4_ due to the work function adjustment by the Mo doping.

Supplementary Fig. 5 shows OCP changes of the Mo doped BiVO_4_ (0.05, 0.1, and 0.5%) relative to the pure BiVO_4_ photoanode due to the truncation of Fermi-level pinning and surface trap states of BiVO_4_. OCP is essentially the difference between open-circuit voltage in dark (OCV_dark_) and light (OCV_light_). The work function is tuned in such a way that the Fermi level (E_f_) is far from CBM when light is off resulting in a small OCV_dark_, but when light is on (AM 1.5 G), the quasi-Fermi-level (E_fn_) becomes as close as possible to CBM giving rise to a large OCV_light_. Consequently, a large OCP (V_ph_) can be obtained. Shown in Supplementary Fig. 5 are the OCP timing profiles of the Mo doped BiVO_4_ (0.05, 0.1, and 0.5%) and the pure BiVO_4_ photoanodes in PPB solution with the Na_2_SO_3_ hole scavenger (pH 7) over a testing interval of 30 s (Supplementary Fig. 5a) and 2000 s (Supplementary Fig. 5b). The most important observation is that the 0.1% Mo-BiVO_4_ photoanode achieved the highest OCP (~1 V in the first irradiation on/off cycle, and ~0.55 V in the cycles after testing for 1600 s). These OCP values are much higher than those of pure-BiVO_4_ (~0.35 V and ~0.15 V), 0.05% Mo-BiVO_4_ (~0.8 V and ~0.4 V), and 0.5% Mo-BiVO_4_ photoanode (~0.23 V and ~0.16 V) when tested under otherwise the same conditions. A higher OCP value means a more favorable driving force for water oxidation since it determines the difference between the hole quasi-Fermi-level of the semiconductor heterojunction and the redox potential of the electrolyte.

The above presented OCP result can be captured by the picture illustrated in Fig. 2b. For the pure BiVO_4_, the Fermi-level position of ideal BiVO_4_ is close the valence band edge of BiVO_4_^9^, but the lattice defects formed during synthesis and surface state trapped electrons (V^4+^) of pure BiVO_4_ move the E_f_ of pure BiVO_4_ negatively (Supplementary Fig. 23)^38–40^. When the pure BiVO_4_ photoanode was immersed in the solution, the E_f_ of pure BiVO_4_ became more negative than the redox potential due to Fermi-level pinning by surface trapped electrons, making the OCV_dark_ of BiVO_4_ relatively high^11^. Meanwhile, under AM 1.5 G illumination, the Fermi-level pinning effect prevented the E_fn_ moving very close to the conduction band minimum (CBM) of BiVO_4_^11,38^, leading to a moderate OCV_light_ and thus a low OCP (V_ph_) of pure BiVO_4_ very low. Importantly, the Mo doping in BiVO_4_ could reduce the surface trap states and at the same time introduced new states, thus moderating the Fermi-level pinning effect (Supplementary Fig. 4)^41^. Due to the reduced Fermi-level pinning effect by Mo doping, for the 0.05% and 0.1% Mo-BiVO_4_ photoanodes, the E_f_ and E_fn_ became more positive and negative than E_f_ of the pure BiVO_4_ photoanode, respectively, thereby enhancing the OCPs. However, for the 0.5% Mo-BiVO_4_, new states were introduced due to the excess Mo doping, and the E_f_ became much closer to CBM, leading to high OCV_dark_ (Supplementary Fig. 6) and thus a low OCP. As such, the OCP of 0.1% Mo-BiVO_4_ is the best of all the samples we studied, in agreement with the corresponding PEC performance as will be presented below. As can be seen from Fig. 2a, the photocurrent density of 0.1% Mo-BiVO_4_ reached 5.0 (±0.2) mA cm^−2^ at 1.23 V vs. RHE in solution with hole scavenger, which represents ~73% the theoretical water oxidation photocurrent density (*J*_max_) of BiVO_4_ (6.8 mA cm^−2^). Thus the moderate Mo doping of BiVO_4_ in the low end doping regime can increase the photo-voltage carrier concentration and mobility of the BiVO_4_ photoanodes, and improve their onset potential and photocurrent density.

### Heterojunction engineering

**Fig. 3:**
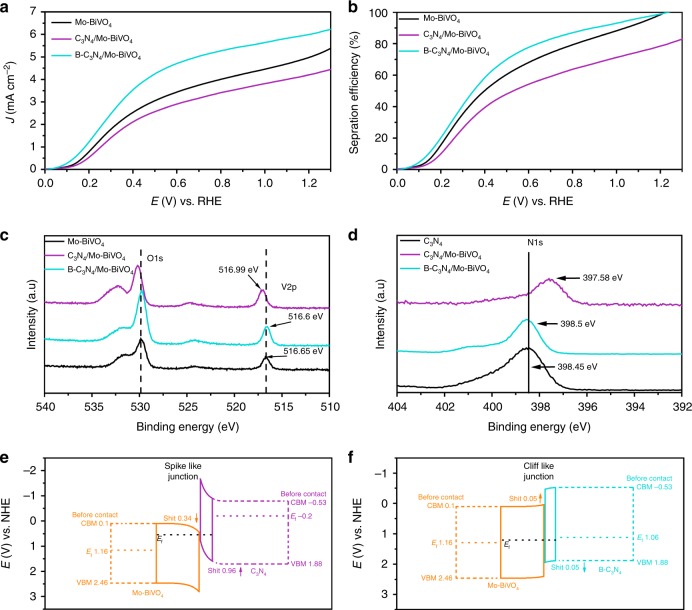
Photoelectrochemical performances and charge transfer processes. **a** LSV curves of C_3_N_4_/Mo-BiVO_4_, 0.1% Mo-BiVO_4_ and B-C_3_N_4_/Mo-BiVO_4_ measured at a scan rate of 25 mV s^−1^ under AM 1.5 G irradiation in PPB solution with Na_2_SO_3_ as a hole scavenger (pH 7). **b** Separation efficiency (*Φ*_Sep_) of C_3_N_4_/Mo-BiVO_4_, 0.1% Mo-BiVO_4_ and B-C_3_N_4_/Mo-BiVO_4_. XPS core-level shifts of **c** V 2p, O 1 s and **d** N 1 s. Schematic diagrams of the band structures of **e** C_3_N_4_/Mo-BiVO_4_, **f** B-C_3_N_4_/Mo-BiVO_4_

We now turn to our second strategy to optimize the PEC performance of BiVO_4_-based photoanodes by further increasing the utilization of *J*_max_. To accomplish it, we started with the basic C_3_N_4_/BiVO_4_ junction, and then work up for optimization by B-doping C_3_N_4_ and the Mo-doping BiVO_4_. Both LHE and charge separation have been enhanced, leading to the increase of *J*_abs_ and *Φ*_Sep_ of the photoanode. Supplementary Fig. 7 shows the UV–vis absorption spectra from the diffuse reflectance measurements and photographs of B-C_3_N_4_ and C_3_N_4_. From visual inspection, the yellow color of B-C_3_N_4_ is clearly deeper than C_3_N_4_, and correspondingly, the absorption of B-C_3_N_4_ is also stronger than C_3_N_4_. From the plots in Supplementary Fig. 8a of (αhν)^2^ vs. the photon energy (hν), the band-gap energy of C_3_N_4_ and B-C_3_N_4_ are 2.53 eV and 2.41 eV, respectively. Supplementary Fig. 8b is the LHE of B-C_3_N_4_/Mo-BiVO_4_, which exhibits stronger absorption in the range between 300 nm and 500 nm than Mo-BiVO_4_. Supplementary Fig. 8c shows the spectra of the solar irradiance of AM 1.5 G (ASTM G173-03) and those weighted by the LHE spectra of B-C_3_N_4_/Mo-BiVO_4_, which shows the *J*_abs_ of B-C_3_N_4_/Mo-BiVO_4_ achieved ~6.0 mA cm^−2^. Figure 3a compares the LSV curves of 0.1% Mo-BiVO_4_ (black line), C_3_N_4_/Mo-BiVO_4_ (cyan line) and B-C_3_N_4_/Mo-BiVO_4_ (purple line) at a scan rate of 25 mV s^−1^ using 0.5 M Na_2_SO_3_ as a hole scavenger in a PPB buffered aqueous solution (pH7) under AM 1.5 G irradiation. The photocurrent density of B-C_3_N_4_/Mo-BiVO_4_ reached ~6 mA (±0.2) cm^−2^ at 1.23 V vs. RHE, and this is higher than that of 0.1% Mo-BiVO_4_ (5 mA cm^−2^), at least partly due to the increase in LHE and *J*_abs_ after the conjugation with B-C_3_N_4_. At 0.6 V vs. RHE, the photocurrent density of B-C_3_N_4_/Mo-BiVO_4_ was 4.7 mA cm^−2^, which is much higher than that of 0.1% Mo-BiVO_4_ (3.4 mA cm^−2^). However, the photocurrent of C_3_N_4_/Mo-BiVO_4_ only achieved 2.9 and 4.2 mA cm^−2^ at 0.6 and 1.23 V vs. RHE, which are lower than that of Mo-BiVO_4_ (3.4 and 5 mA cm^−2^), although increased LHE and *J*_abs_ by compositing C_3_N_4_ and B-C_3_N_4_. The results of *Φ*_Sep_ of Mo-BiVO_4_ (black line), C_3_N_4_/Mo-BiVO_4_ (cyan line) and B-C_3_N_4_/Mo-BiVO_4_(purple line) clearly shows that B-C_3_N_4_ decoration can great increase the *Φ*_Sep_ of Mo-BiVO_4_, while pure C_3_N_4_ decoration will reduce the *Φ*_Sep_ of Mo-BiVO_4_ (Fig. 3b). The *Φ*_Sep_ of B-C_3_N_4_/Mo-BiVO_4_ achieved 79 and 98% at 0.6 V and 1.23 V vs. RHE, respectively. The *Φ*_Sep_ of B-C_3_N_4_/Mo-BiVO_4_ at 0.6 V vs. RHE is higher than that of Mo-BiVO_4_ (69%), and at 1.23 V vs. RHE, the *Φ*_Sep_ of Mo-BiVO_4_ andB-C_3_N_4_/Mo-BiVO_4_ are achieved near 100% cause the charge separation of Mo-BiVO_4_ and B-C_3_N_4_/Mo-BiVO_4_ have reached their limits at higher bias voltage. However, the *Φ*_Sep_ of C_3_N_4_/Mo-BiVO_4_ only achieved 60 and 80% at 0.6 V and 1.23 V vs. RHE, which are even lower than that of Mo-BiVO_4_. This result shows that when C_3_N_4_ is compositing the Mo-BiVO_4_, it has no effect on the separation of the photo-generated charges. These results mean that although the thermodynamic potential of pure C_3_N_4_ and Mo-BiVO_4_ are match, the heterojunction of pure C_3_N_4_ and Mo-BiVO_4_ became a compound center of photo-generated charge.

As shown in Fig. 3c, d, when the Mo-BiVO_4_ and pure C_3_N_4_ are in contact, the O1s and V2p positive shift 0.34 eV and N1s negative shifts 0.96 eV. Supplementary Fig. 9 shows the CBM, VBM and Fermi level of BiVO_4_, Mo-BiVO_4,_ C_3_N_4_ and B-C_3_N_4_ by UPS and XPS VB spectra data (Supplementary Figs. 10, 11, 12, and 13). The Fermi level of BiVO_4_ and Mo-BiVO_4_ are in the middle of their band gap (BiVO_4_: 1.21 eV and Mo-BiVO_4_: 1.16 eV) and the Fermi level of B-C_3_N_4_ is close to its VBM, and the Fermi level of pure C_3_N_4_ is close to its CBM. The DFT data confirms that the Fermi energy level of Mo-BiVO_4_ and B-C_3_N_4_ changed after Mo and B element doping (Supplementary Fig. 14). In detail, when Mo-BiVO_4_ and C_3_N_4_ come into contact to form a heterojunction, the bands on the two sides bend oppositely into the spike-like structure (Fig. 3e), and thus the electrons from the C_3_N_4_ side can hardly transfer to the Mo-BiVO_4_ side. The holes inside Mo-BiVO_4_ will hardly transfer to C_3_N_4_, as the existence of energy barrier at the interface. Therefore, the contact interface of pure C_3_N_4_ and Mo-BiVO_4_ will reduce the separation of photo-generated charges. On the other hand, due to the B element doping, the Fermi level of B-C_3_N_4_ is getting closer to the VBM (Supplementary Fig. 9b). As shown in Fig. 3d, when the Mo-BiVO_4_ and B-C_3_N_4_ are in contact, a cliff like junction is formed with the correct charge transfer direction, which will increase the separation of photo-generated charges. Supplementary Fig. 15 displays the Raman spectra of Mo-BiVO_4_, C_3_N_4_, B-C_3_N_4_, C_3_N_4_/Mo-BiVO_4_ and B-C_3_N_4_/Mo-BiVO_4_. The Raman peaks at 702 cm^−1^ and 746 cm^−1^ are attributed to the C-N vibration of B-C_3_N_4_ (Blue line). The peak at 816 cm^−1^ are attributed to the V–O vibration, while the peaks at 362 cm^−1^ and 323 cm^−1^ can be attributed to the VO_4_^3-^ vibration of the Mo-BiVO_4_. The Raman peaks of B-C_3_N_4_ (702 cm^−1^, 746 cm^−1^) and Mo-BiVO_4_ (362 cm^−1^, 323 cm^−1^) shifted to 545 cm^−1^, 620 cm^−1^, and 340 cm^−1^, 314 cm^−1^ in B-C_3_N_4_/Mo-BiVO_4_ sample, indicating the formation of chemical bonds between B-C_3_N_4_ and Mo-BiVO_4_. In other words, the B-C_3_N_4_ is chemically linked to Mo-BiVO_4_.

### PEC water splitting performance

**Fig. 4:**
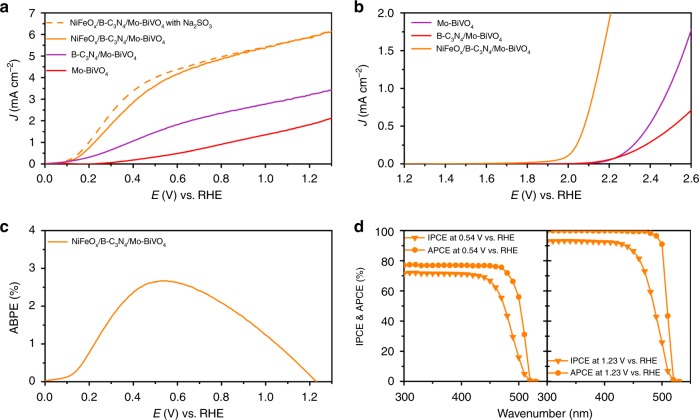
Photoelectrochemical performances. LSV curves of the Mo-BiVO_4_, B-C_3_N_4_/Mo-BiVO_4_ and NiFeO_x_/B-C_3_N_4_/Mo-BiVO_4_ recorded at a scan rate of 25 mV s^−1^ in PPB solution without Na_2_SO_3_ as a hole scavenger (pH 7) **a** under AM 1.5 G irradiation and **b** in dark. **c** ABPE of NiFeO_x_/B-C_3_N_4_/Mo-BiVO_4_. **d** IPCE and APCE of NiFeO_x_/B-C_3_N_4_/Mo-BiVO_4_ at 0.54 V vs. RHE (left) and 1.23 V vs. RHE (right) in PPB solution (pH 7)

In order to use the B-C_3_N_4_/Mo-BiVO_4_ photoanode for PEC water splitting in solution without hole scavenger, the NiFeO_x_ layer was used as an OEC material, which was deposited on the photo-active area of B-C_3_N_4_/Mo-BiVO_4_ photoanode surface by photoelectrodeposition method. Supplementary Figs. 16 and 17 show the XPS data of Ni, N, B, C, Bi, Mo, O, and Fe. For B-doped C_3_N_4_, as can be seen from Supplementary Figs. 16c, the peak of B is located at around 191.9 eV, fairly close to the binding energy of B in the –C–N–B– and –N–B–(N)_2_– groups (192.1 eV) of the B-C_3_N_4_ materials reported in the literature^42^. The oxidation state of B is consistent with the results of B-doped C_3_N_4_ in the reported literature^27^. As shown in Fig. 4a and Supplementary Fig. 21, the PEC water splitting capability of NiFeO_x_/B-C_3_N_4_/Mo-BiVO_4_ photoanode (orange solid line) achieves 4.18 mA cm^−2^ and 5.93 (±0.3) mA cm^−2^ at 0.6 V vs. RHE and 1.23 V vs. RHE in PPB solution (pH 7), which are much higher than that of Mo-BiVO_4_ photoanode and B-C_3_N_4_/Mo-BiVO_4_, Meanwhile, the photocurrent density of NiFeO_x_/B-C_3_N_4_/Mo-BiVO_4_ photoanode at 0.6 V vs. RHE and 1.23 V vs. RHE are much close to that of NiFeO_x_/B-C_3_N/Mo-BiVO_4_ photoanode (orange dotted line) in PPB solution with Na_2_SO_3_ hole scavenger (pH 7, 4.37 (±0.3) mA cm^−2^ and 5.96 (±0.3) mA cm^−2^). Supplementary Fig. 18 shows that the B-C_3_N_4_ can not only increase the photocurrent density in the low biased-voltage range (0.1~0.8 V vs. RHE) by increasing the separation efficiency of photoanode, but also increase the photocurrent density in the high biased-voltage range (0.8~1.3 V vs. RHE). The IPCEs of NiFeO_x_/B-C_3_N_4_/Mo-BiVO_4_, NiFeO_x_/Mo-BiVO_4_, NiFeO_x_/ BiVO_4_, B-C_3_N_4_/Mo-BiVO_4_, Mo-BiVO_4_, and BiVO_4_ at 0.54 V vs. RHE in PPB solution (pH 7), which shows that the B-C_3_N_4_ can increase the charge separation efficiency and light absorption of photoanode. On the other hand, the NiFeO_x_ cannot increase light absorption of photoanode, and it only plays the role of a co-catalyst (OEC) here (Supplementary Fig. 19). In Fig. 4b, due to the high oxygen evolution reaction capacity of NiFeO_x_ OEC, the dark LSV of NiFeO_x_/B-C_3_N/Mo-BiVO_4_ photoanode shows a remarkable cathodic shift (~340 mV) of onset potential compared to B-C_3_N_4_/Mo-BiVO_4_ photoanode and Mo-BiVO_4_ photoanode. However, the LSV curves of the B-C_3_N_4_/Mo-BiVO_4_ and NiFeO_x_/B-C_3_N_4_/Mo-BiVO_4_ recorded at a scan rate of 25 mV s^−1^ in PPB solution with Na_2_SO_3_ as a hole scavenger (pH 7), which shows that the photocurrent density of samples measured in solution with Na_2_SO_3_ will decline after the NiFeO_x_ layer deposition (Supplementary Fig. 20). Figure 4c shows the half-cell applied bias photo-to-current efficiency (ABPE) of the NiFeO_x_/B-C_3_N_4_/Mo-BiVO_4_ photoanode. The ABPE is calculated to be 2.67% at 0.54 V vs. RHE, which is the highest recorded for BiVO_4_-based photoanodes (Fig. 6c). Evidently, the highest efficiency has been achieved for the NiFeO_x_/B-C_3_N_4_/Mo-BiVO_4_ photoanode at the lowest potential (0.54 V vs. RHE) among the previously reported values, which shows the highest performance of PEC water splitting for BiVO_4_-based photoanodes. Shown in Supplementary Figs. 25 and 26 are the LSV curves and ABPE measurement results in the two electrodes configuration (NiFeO_x_/B-C_3_N_4_/Mo-BiVO_4_ photoanode and Pt cathode) in PPB solution (pH 7). It can be seen that the ABPE of whole PEC system (without reference electrode) achieved 2.1% at 0.62 V vs. Pt. Figure 4d shows the incident-photon-to-current conversion efficiency (IPCE) and the absorbed photon-to-current efficiency (APCE) spectra of NiFeO_x_/B-C_3_N_4_/Mo-BiVO_4_ photoanode at 0.54 V *vs*. RHE and 1.23 V vs. RHE in PPB solution (pH 7). The maximum IPCE value of NiFeO_x_/B-C_3_N_4_/Mo-BiVO_4_ photoanode reaches ~71 and 92% at 0.54 V vs. RHE and 1.23 V vs. RHE. The APCE of NiFeO_x_/B-C_3_N_4_/Mo-BiVO_4_ photoanode reaches ~100% at 1.23 V vs. RHE, establishing the NiFeO_x_/B-C_3_N_4_/Mo-BiVO_4_ photoanode used all the light it could absorb for PEC water splitting at 1.23 V vs. RHE. Therefore, these results clearly show that the NiFeO_x_ OEC can greatly improve the PEC capacity of B-C_3_N_4_/Mo-BiVO_4_ photoanode in the absence of any hole scavenger.

**Fig. 5:**
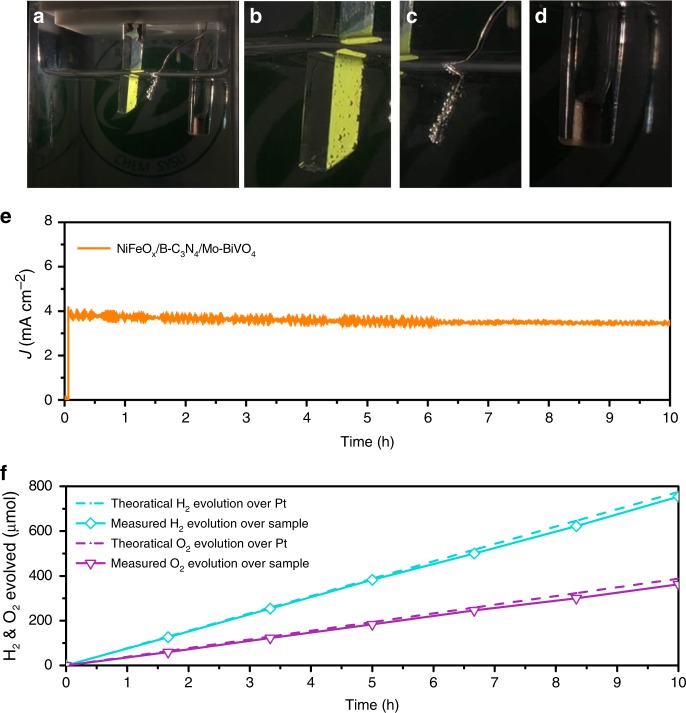
Photoelectrochemical water splitting performances. Photographs of the NiFeO_x_/B-C_3_N_4_/Mo-BiVO_4_ photoanode in the PEC water splitting system (**a**), the NiFeO_x_/B-C_3_N_4_/Mo-BiVO_4_ photoanode in a blown-up view (**b**), the Pt cathode (**c**), and the Ag/AgCl reference electrode (**d**). **e** Chronoamperometry (i–t) curve of NiFeO_x_/B-C_3_N_4_/Mo-BiVO_4_ photoanode collected at 0.54 V vs. RHE under AM 1.5 G illumination in PPB solution (pH 7). **f** H_2_ and O_2_ evolution of the NiFeO_x_/B-C_3_N_4_/Mo-BiVO_4_ photoanode at 0.54 V vs. RHE; dashed curves indicate the H_2_ and O_2_ evolution with 98% Faraday efficiency

As shown in Fig. 5 and Supplementary Video 1, the NiFeO_x_/B-C_3_N/Mo-BiVO_4_ photoanode exhibits excellent operational stability for half-cell PEC water splitting in PPB solution (pH 7) at 0.54 V vs. RHE under AM 1.5 G irradiation. Figure 5a shows the photograph of the NiFeO_x_/B-C_3_N/Mo-BiVO_4_ photoanode half-cell PEC water splitting system, and Fig. 5b–d show the photographs of the photoanode, the Pt cathode and the Ag/AgCl reference electrode separately. The chronoamperometry curve of the NiFeO_x_/B-C_3_N_4_/Mo-BiVO_4_ photoanode was collected at 0.54 V vs. RHE in 10 h. The photocurrent density of the NiFeO_x_/B-C_3_N_4_/Mo-BiVO_4_ photoanode was initially 3.85 mA cm^−2^ and decreased by only 10% after 10 h of operation, demonstrating the good stability of the NiFeO_x_/B-C_3_N_4_/Mo-BiVO_4_ photoanode during the long time irradiation in PPB solution (Fig. 5e). And the photocurrent densities of the NiFeO_x_/Mo-BiVO_4_ and NiFeO_x_/B-C_3_N_4_/Mo-BiVO_4_ photoanodes decayed by 5 and 8% (Supplementary Fig. 24), respectively, very close to the stability testing result of the NiFeO_x_/B-C_3_N_4_/Mo-BiVO_4_ photoanode at 0.54 V vs. RHE. These results confirm that the NiFeO_x_ is a stable co-catalyst for the BiVO_4_-based photoanodes, essentially consistent with the recent reports. The generation rates of H_2_ and O_2_ by our half-cell system are measured to be 77.5 μM/h and 336 μm 33.6 μM/h, respectively, with Faradic efficiency of 98% (Fig. 5f).

## Discussion

**Fig. 6:**
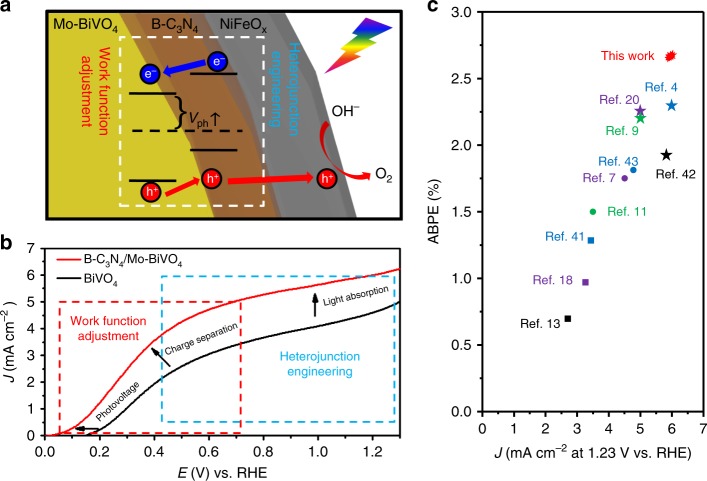
Photoelectrochemical water splitting characterization. **a** Schematic diagram illustrating the separation of photo-generated electrons and holes of the NiFeO_x_/B-C_3_N_4_/Mo-BiVO_4_ photoanode, **b** LSV curves of pure-BiVO_4_, B-C_3_N_4_/Mo-BiVO_4_ recorded at a scan rate of 25 mV s^−1^ under AM 1.5 G one-sun irradiation in PPB solution using 0.5 M Na_2_SO_3_ as a hole scavenger (pH 7), **c** specific photocurrent density at 1.23 V vs. RHE and applied bias photo-to-current efficiency (ABPE) of BiVO_4_ based photoanode^4,7,9,11,13,18,20,41–43^.

In conclusion, the NiFeO_x_/B-C_3_N_4_/Mo-BiVO_4_ photoanode has provided an archetype to exploit the potential of boosting the photoelectrochemical performance by the synergistic combination of work function tuning and heterojunction construction. The bespoke photoanode achieved a remarkable photocurrent density of 3.85 mA cm^−2^, ABPE of 2.67% and IPCE of 71% at 0.54 V vs. RHE, which are the highest yet reported with the lowest biased-voltage for BiVO_4_-based PEC materials. The NiFeO_x_/B-C_3_N/Mo-BiVO_4_ photoanode exhibited significantly enhanced PEC activity for water splitting by systematically work function adjustment (Fig. 6a). We have demonstrated the work function adjustment via Mo doping could reduce the interfacial energy loss and increase the open-circuit photo-voltage of BiVO_4_ PEC cells. In addition, the creation and optimization of the heterojunction (p-n) of B-C_3_N_4_ and Mo-BiVO_4_ with correct charge transfer direction were accomplished by work function adjustment via B doping for C_3_N_4_, thereby increasing the separation of photo-generated electron-hole pairs at the B-C_3_N_4_ and Mo-BiVO_4_ interface (Fig. 6a, b). The data of DFT calculation, XPS and UPS confirm the Fermi level and band shift of B-C_3_N_4_ and Mo-BiVO_4_. This synergistic effect between B doping of C_3_N_4_ and Mo doping of BiVO_4_ with the NiFeO_x_ OEC has allowed the NiFeO_x_/B-C_3_N_4_/Mo-BiVO_4_ photoanode to achieve the record – the highest PEC water splitting performance (2.67% ABPE) with a fairly low bias-voltage (0.54 V vs. RHE), which shows the efficiency of ABPE are the highest recorded for BiVO_4_-based photoanodes^4,7,9,11,13,18,20,43–45^. The demonstration of the NiFeO_x_/B-C_3_N_4_/Mo-BiVO_4_ photoanode with excellent PEC water splitting capability achieved by the synergistic combination of work function tuning and heterojunction deliberation will inform the design and development of the next-generation PEC materials and devices.

## Methods

### Preparation of BiVO_4_ and Mo-BiVO_4_ electrode

BiVO_4_ photoanodes were fabricated by a two-step process via a modified method which was originally developed by Kim and Choi^7^. At first, a template-free electrochemical deposition was applied to prepare the BiOI nanosheets using a conventional three-electrode glass cell, where a piece of F-doped SnO_2_ coated glass (FTO, Nippon Sheet Glass, 1 × 2 cm) served as the working electrode, a Pt electrode served as the counter electrode and an Ag/AgCl electrode served as the reference electrode. Generally, 50 mL of solution containing 0.4 M KI (99.0%, Tianjin Zhiyuan Reagent Co. Ltd.) and 0.04 M Bi(NO_3_)_3_ (99.0%, Shanghai Macklin Biochemical Co. Ltd) was adjusted pH to 1.7 by adding HNO_3_ (65–68%, AR, Guangzhou Chemical Reagent). Then, 20 mL of absolute ethanol (100%) containing 0.23 M p-benzoquinone (97%, Aladdin) was mixed into the above solution and vigorously stirred for several minutes. Cathodic deposition of BiOI (1 × 1 cm area) was performed potentiostatically in the final solution at −0.1 V vs. Ag/AgCl at room temperature (RT) for 200 s. The second step was the conversion of BiOI to BiVO_4_. Dimethyl sulfoxide (DMSO, AR, Tianjin Damao Reagent) solution containing 0.2 M vanadyl acetylacetonate (VO(acac)_2_, 95%, Aladdin) was impregnated on BiOI electrodes (50 μL cm^−1^) and then annealed in air at 450 °C for 2 h with ramping rate of 2 °C min^−1^. Lastly, the BiVO_4_ electrodes were soaked in 1 M NaOH (AR, Guangzhou Chemical Reagent) solution for 1 h with gentle stirring to remove the excess V_2_O_5_. The obtained pure BiVO_4_ electrodes were rinsed by deionizer water and dried at RT.

Molybdenum doped BiVO_4_ (Mo-BiVO_4_) photoanodes were prepared in the same way by adding Na_2_MoO_4_ as the Mo source. In detail, 1, 2, and 10 μL 0.1 M Na_2_MoO_4_ (AR, Tianjin Damao Reagent) aqueous solution was added into 1 mL the above VO(acac)_2_ DMSO solution before it was impregnated on BiOI electrodes. The corresponding concentration ratio of Mo/Bi was 0.05%, 0.1%, and 0.5%, respectively.

### Preparation of C_3_N_4_ and B-C_3_N_4_

The bulk graphite-C_3_N_4_ (C_3_N_4_) was fabricated by directly heating low-cost melamine (99%, Aladdin). In detail, 5 g melamine powder was placed in an alumina crucible with a cover, then heated to 500 °C for 2 h in a muffle furnace with a heating rate of 2 °C min^−1^. The obtained bulk C_3_N_4_ was grind into small powder, and 100 mg C_3_N_4_ powder was dispersed in 100 mL isopropyl alcohol (AR, Tianjin Damao Reagent) and exfoliated by ultrasonication for 24 h to obtain C_3_N_4_ nanosheets (C_3_N_4_-NS). The resultant dispersion was centrifuged at 3000 rpm for 10 min, and the supernatant containing exfoliated C_3_N_4_-NS was collected by pipette. Boron doped C_3_N_4_ nanosheets (B-C_3_N_4_-NS) supernatants were prepared in the same way but heating the mixture of 0.5 g boric acid (GR, Aladdin) and 5 g melamine.

### Preparation of C_3_N_4_/BiVO_4_ and B-C_3_N_4_/Mo-BiVO_4_ electrode

The BiVO_4_ or Mo-BiVO_4_ photoanodes were immersed into 20 mL C_3_N_4_-NS or B-C_3_N_4_-NS supernatants for 1 h. After rinsed with deionizer water, the obtained (B-)C_3_N_4_/(Mo-)BiVO_4_photoanodes were annealed at 350 °C in air for 1 h for better combination.

### Photoelectrodeposition of NiFeO_x_ OEC layer

NiFeO_x_ OEC layer were synthesized using a simple photoelectrodeposition method reported. NiFeO_x_ layer was photoelectrodeposited on the B-C_3_N_4_/Mo-BiVO_4_ in 0.4 M FeSO_4_ and 0.04 M NiSO_4_ solution at 0.6 V vs. Ag/AgCl (total charge 100 mC/cm^2^) at RT. The NiFeO_x_/B-C_3_N_4_/Mo-BiVO_4_ electrode was then washed with deionizer water and dried in air at 60 °C.

### Analysis

The as-synthesized products were characterized by a scanning electron microscope (SEM, Zeiss G-500), transmission electron microscopy (TEM, JEOL 2100 F, FEI Tecnai G^2^F30), X-Ray Diffractometer (XRD, D8 ADVANCE), X-ray Photoelectron Spectroscopy (XPS) and Ultroviolet Photoelectron Spectroscopy (UPS, Thermo Fisher Scientific ESCALab250) and Raman spectroscopy (Renishaw inVia). The optical properties of the products were measured with an UV–vis–NIR Spectrophotometer (UV–vis–NIR, Shimadzu UV-2450).

### Photoelectrochemical and electrochemical measurements

All the PEC and electrochemical measurements were carried out in a three-electrode cell with a flat quartz window to facilitate illumination of the photoelectrode surface. The working electrode is the product fabricated in this work, while Pt electrode was used as a counter electrode and Ag/AgCl electrode was used as a reference electrode, respectively. The illumination source was AM 1.5 G solar simulator (Newport, LCS 100 94011 A (class A, Supplementary Fig. 29) directed at the quartz PEC cell (100 mW cm^−2^). Incident-photon-to-current conversion efficiency (IPCE) were collected by a Solartron 1280B electrochemical station with a solar simulator (Newport 69920, 1000 W xenon lamp), coupled with an infrared water filter (Oriel 6127) and aligned monochromator (Oriel Cornerstone 130 1/8 m). All the electrochemical measurements were performed on an SP-150 electrochemical workstation (SP-150, Bio-Logic SAS, France) at RT. More PEC and electrochemical calculations are shown in the Supporting information.

## Supplementary information

SUPPLEMENTARY INFORMATION

Supplementary Video 1

## Data Availability

The authors declare that the main data supporting the findings of this study are available within the article and its Supplementary Information files. Extra data are available from the corresponding author upon request. All relevant data are available from the authors upon reasonable request.
