# Supplementary material for: Enhancing photoelectrochemical water splitting by combining work function tuning and heterojunction engineering
Source: Nat Commun. 2019 Aug 15;10:3687. doi: 10.1038/s41467-019-11586-y (PMC6695449; doi:10.1038/s41467-019-11586-y)
Supplement: Supplementary file 1 — SUPPLEMENTARY INFORMATION [file 41467_2019_11586_MOESM1_ESM.doc]

**Supplementary Information**

Ye at al.

Enhancing Photoelectrochemical Water Splitting by Combining Work Function Tuning and Heterojunction Engineering

**Supplementary Methods**

**Calculations**

The conversion between potentials *vs.* Ag/AgCl and those *vs.* RHE is performed using the equation below^1^:

E (*vs.* RHE) = E (*vs.* Ag/AgCL) +E_Ag/AgCl_ (reference) +0.0591 V × pH (1)

(E_Ag/AgCl_ (reference) =0.1976 V *vs.* NHE at 25 ^o^C)

Incident-photon-to-current conversion efficiency (IPCE) can be expressed as^2^:

IPCE = (1240 ×*I*) / (λ ×*J*_light_) (2)

where *I* is the photocurrent density, *λ* is the incident light wavelength, and *J*_light_ is the measured irradiance.

Light harvesting efficiency (LHE) can be expressed as^1^:

LHE = 1 - 10^-A(λ)^ (3)

where A_(λ)_ is absorbance, λ is wavelength.

The maximum photocurrent density (*J*_max_)^3^:

Firstly, the National Renewable Energy Laboratory (NREL) reference solar spectral irradiance at AM 1.5G (radiation energy (W·m^-2^·nm^-1^) *vs.* wavelength (nm))was converted to the solar energy spectrum in terms of number of photons (s^-1^·m^-2^·nm^-1^) *vs.* wavelength (nm). Then, the number of photons above the photo-active range of the NiFeO_x_/B-C_3_N_4_/Mo-BiVO_4_ shown in this study (300 nm ~ 530 nm) was calculated using a trapezoidal integration (in 10 nm increments) of the spectrum and was converted to the current density (mA·cm^-2^).

Photocurrent assuming 100% absorbed photon-to-current efficiency (APCE) (*J*_abs_) can be expressed as^1,4^:

(4)

where *I_λ_* is the solar irradiance, *e* is the electron charge, *h* is the Plank constant, *c* is the speed of light, *A* is the absorbance.

The practical water oxidation photocurrent (*J*_PEC_) can be expressed as^1,4^:

*J*_PEC_ ≈ *J*_abs_× Φ_sep_ × Φ_OX_ (5)

The electron-hole separation yield (Φ_sep_) can be expressed as^1,4^:

Φ_sep_ = *J*_HS_ / *J*_abs_ (6)

where *J*_HS_ is the photocurrent density of sample with Na_2_SO_3_ hole scavenger.

Yield of the surface reaching holes or be named transfer efficiency (Φ_OX_) can be expressed as^1,4,5^:

Φ_OX_ = (*J*PEC) / (*J*HS) (7)

Applied bias photo-to-current efficiency (ABPE) can be expressed as^6^:

ABPE = [*J*_PEC_× (1.23 - *V*_app_)] / *P*_light_ (8)

where *J*_PEC_ is the photocurrent density of samples, *V*_app_ is the applied external potential *vs.* RHE and *P*_light_ is the power density of the illumination (100mWcm^-2^).

**DFT Calculations**

All calculations have been carried out with the Vienna ab initio Simulation Package (VASP)^4,7-11^. 3D periodic boundary conditions were applied to simulate the infinitely large systems. A 20 Å vacuum space between sheets was set to prevent the interaction between two membrane layers. The Brillouin zone of the fiber was sampled by 1 × 3 × 1 k‐points. The electronic structure of the system was treated using the generalized gradient approximation with the PBE functional^9^. The van der Waals interactions were added to the standard DFT description by Grimme's D2 scheme^10^.

In all calculations the convergence parameters were 10^−6^ eV for the energy, 0.01 eV Å^−1^ for the forces and an energy cut-off of 500 eV. A Gaussian smearing of 0.05 eV was applied. Charge analysis was performed via Bader analysis^11^, which included the core charges, and charge density difference analysis within VASP.


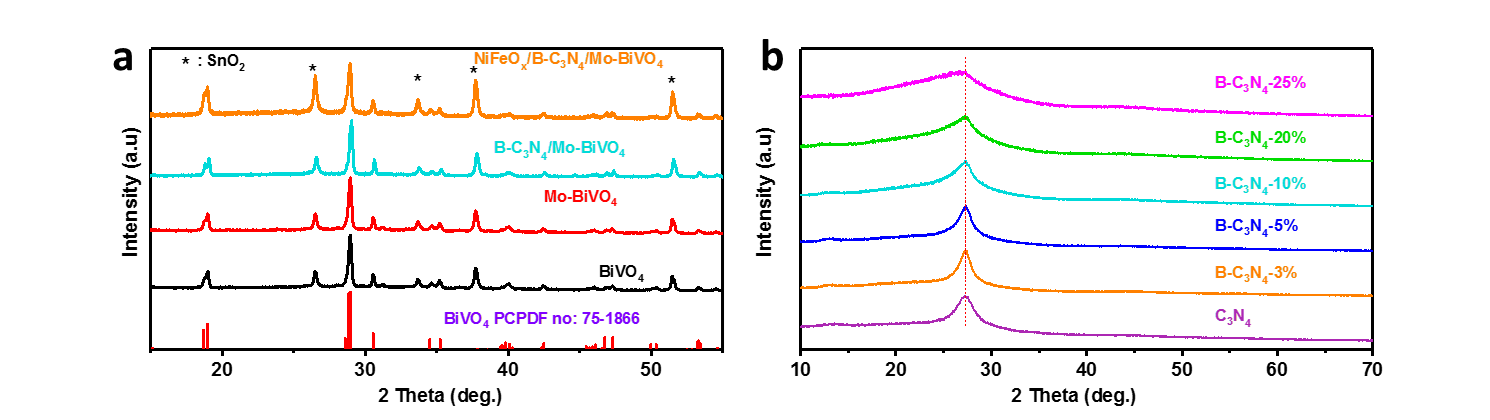


**Supplementary Figure 1.** XRD patterns of the samples. XRD patterns of **a** BiVO_4_, Mo-BiVO_4_, B-C_3_N_4_/Mo-BiVO_4_, and NiFeO_x_/B-C_3_N_4_/Mo-BiVO_4_, **b** C_3_N_4_ and B-C_3_N_4_.


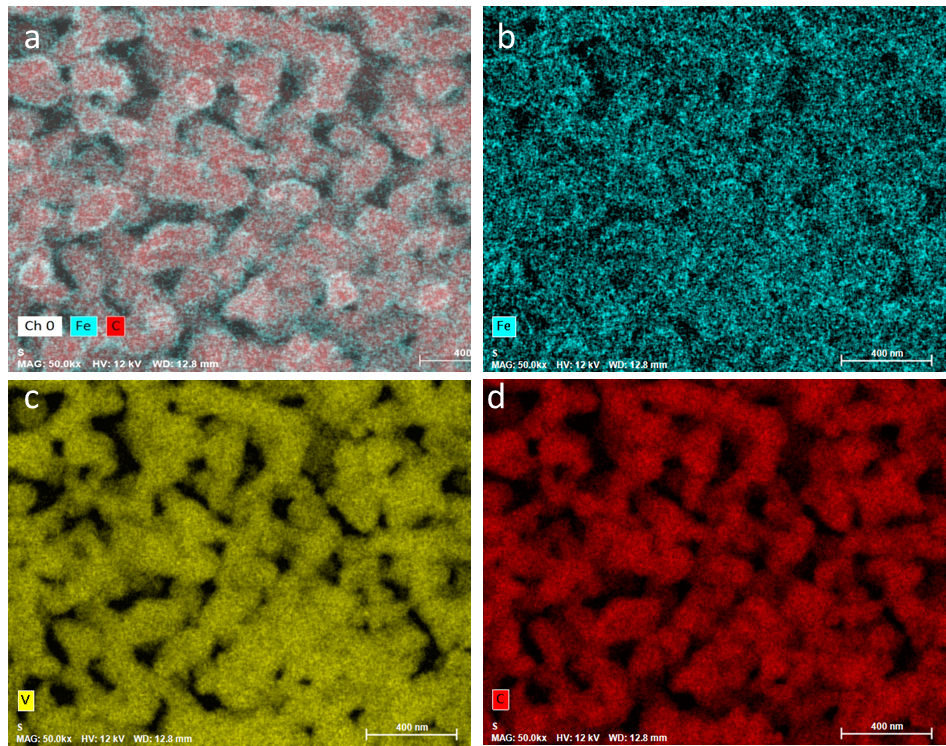


**Supplementary Figure 2.** Elemental mapping images of NiFeO_x_/B-C_3_N_4_/Mo-BiVO_4_. **a** Combined elemental mapping image of Fe and C. **b-d** The corresponding SEM-EDS elemental mapping images for Fe, V and C respectively.


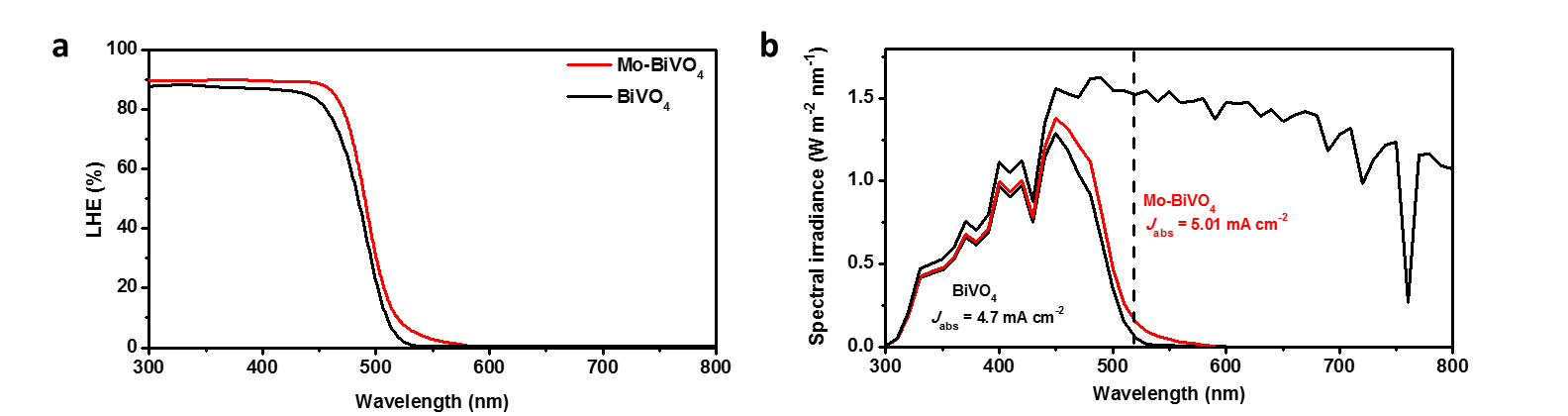


**Supplementary Figure 3.** LHE properties of BiVO_4_ and Mo-BiVO_4_. The **a** LHE spectra and **b** Spectra of the solar irradiance of AM 1.5G (ASTM G173-03) and those weighted by the LHE spectra of the BiVO_4_ and Mo-BiVO_4_.


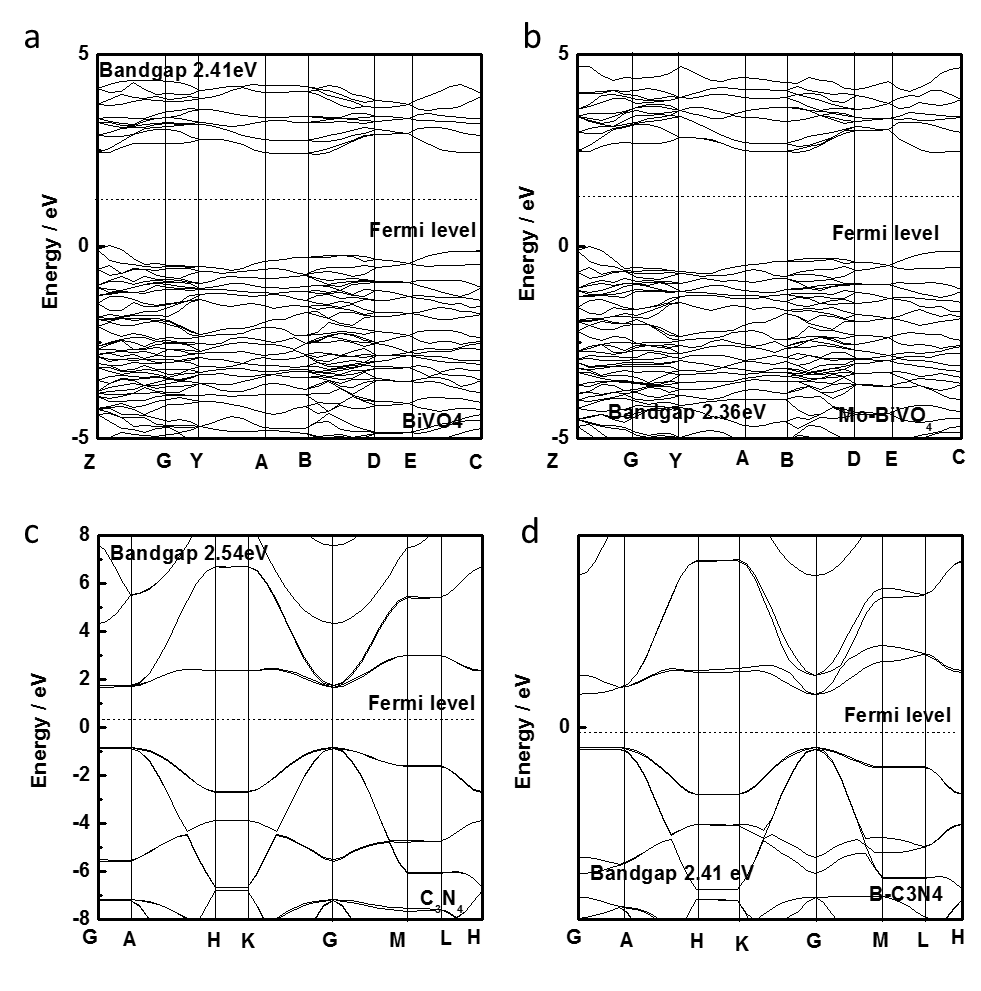


**Supplementary Figure 4.** Band structures. Band structures of **a** BiVO_4_ and **b** Mo-BiVO_4_.


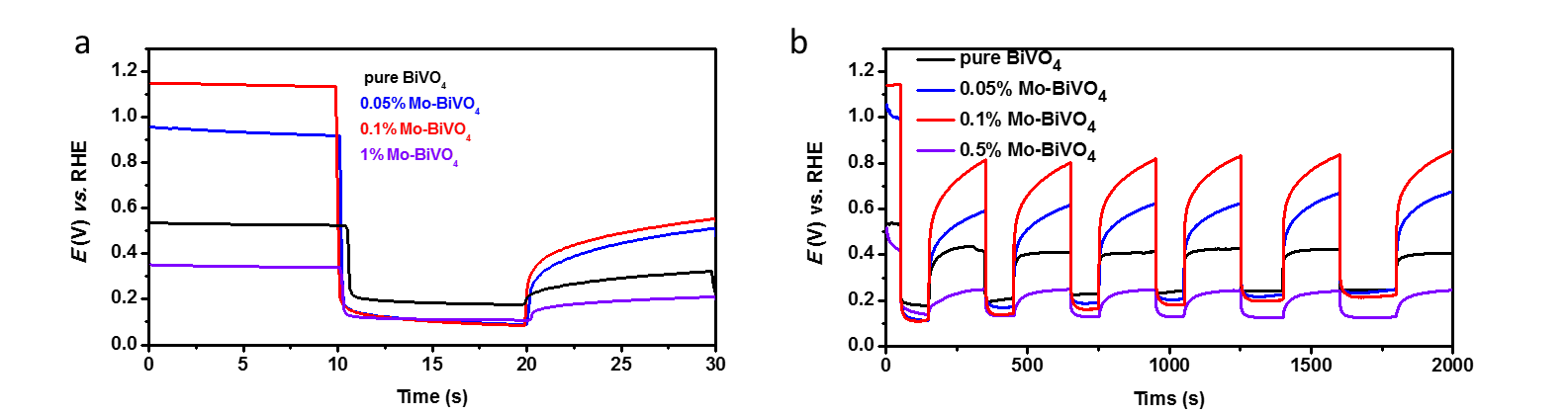


**Supplementary Figure 5.** OCP spectra of BiVO_4_-based photoanodes with hole scavenger. OCP of BiVO_4_, 0.05 Mo-BiVO_4_, 0.1 Mo-BiVO_4_ and 0.5% Mo-BiVO_4_ in PPB solution with the Na_2_SO_3_ hole scavenger (pH = 7) after testing **a** 30 seconds and **b** 2000 seconds.


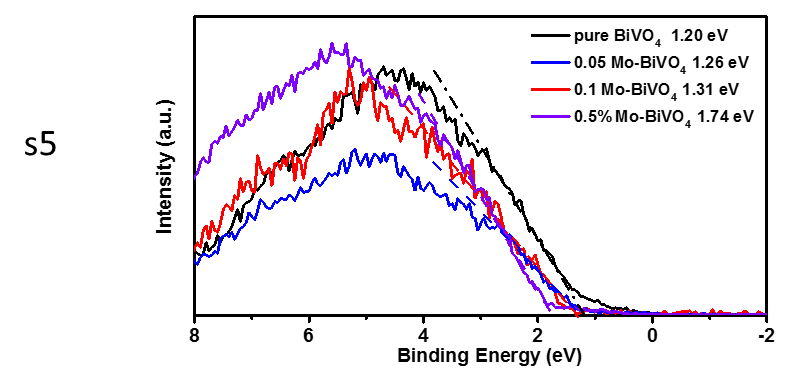


**Supplementary Figure 6.** XPS valence band spectra of BiVO_4_-based photoanodes. XPS valence band spectra of the BiVO_4_, 0.05 Mo-BiVO_4_, 0.1 Mo-BiVO_4_ and 0.5% Mo-BiVO_4_.


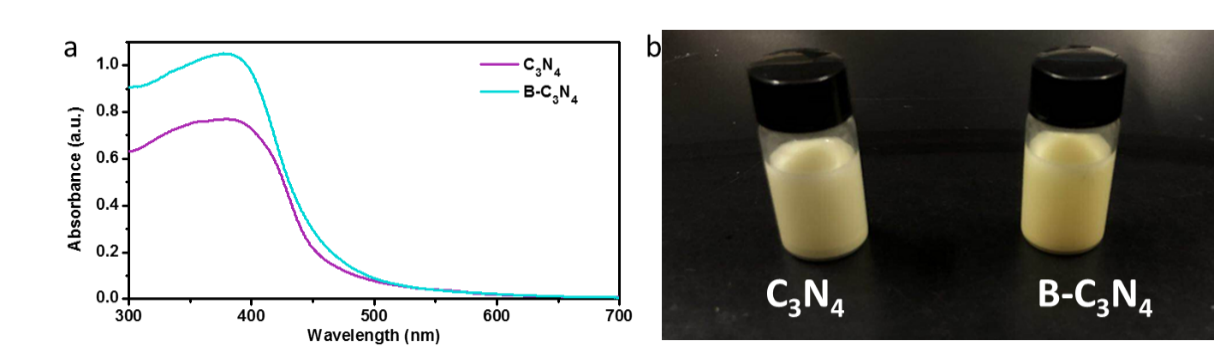


**Supplementary Figure 7.** UV-vis spectra and photo image. **a** UV-vis spectra and **b** photo image of C_3_N_4_ and B-C_3_N_4_.


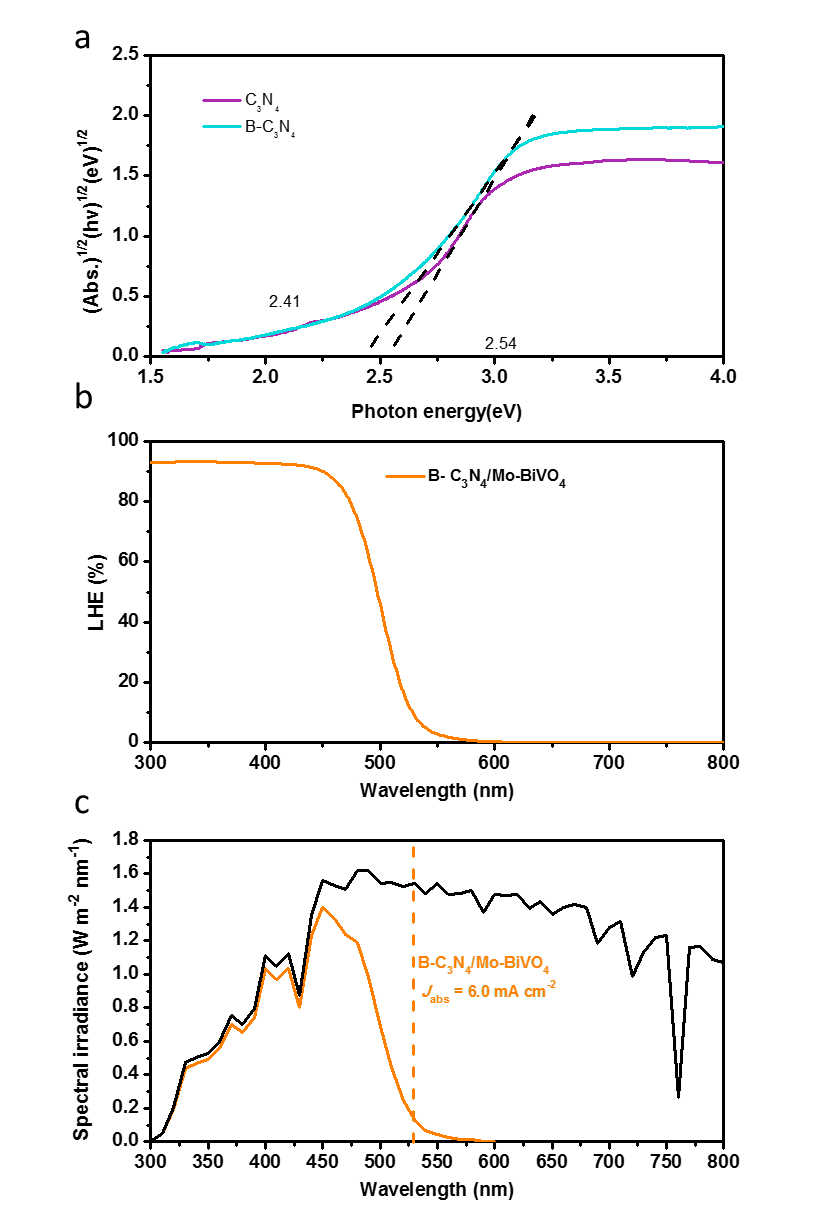


**Supplementary Figure 8.** Optical band gaps or LHE properties of the samples. **a** Plots of (αhν)^2^ *vs.* the photon energy (hν) of the C_3_N_4_ and B-C_3_N_4_, **b** LHE of B-C_3_N_4_/Mo-BiVO_4_ and **c** Spectra of the solar irradiance of AM 1.5G (ASTM G173-03) and those weighted by the LHE spectra of the B-C_3_N_4_/Mo-BiVO_4_.


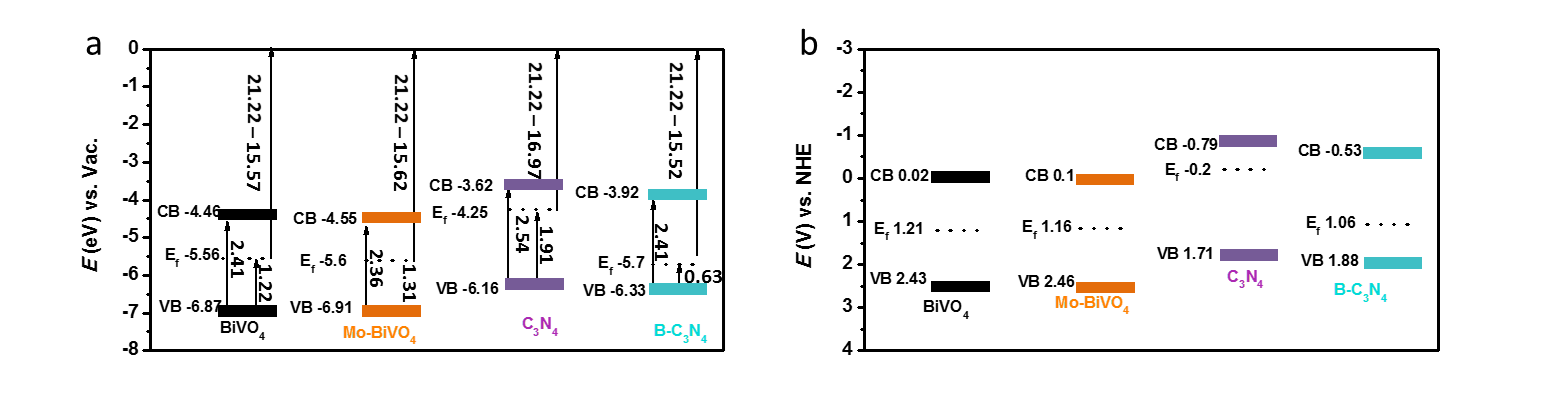


**Supplementary Figure 9.** Schematics of band structures *vs.* Vac. and NHE. Band gap, VBM, CBM and Fermi level of BiVO_4_, Mo-BiVO_4_, C_3_N_4_ and B-C_3_N_4_ *vs.* **a** Vac. and **b** NHE.


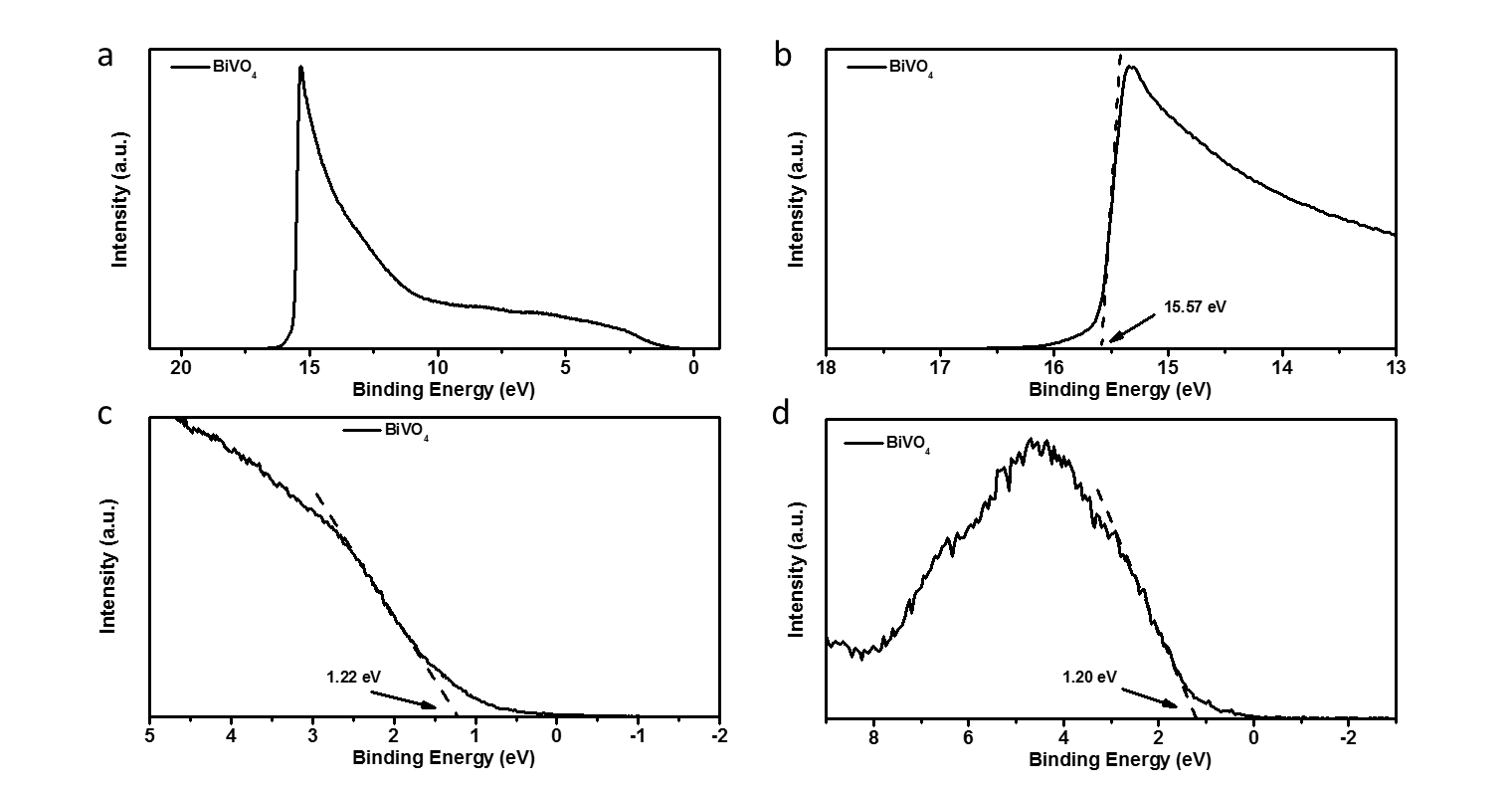


**Supplementary Figure 10.** UPS and XPS valence band spectra**. a-c** UPS and **d** XPS valence band spectra of BiVO_4_.


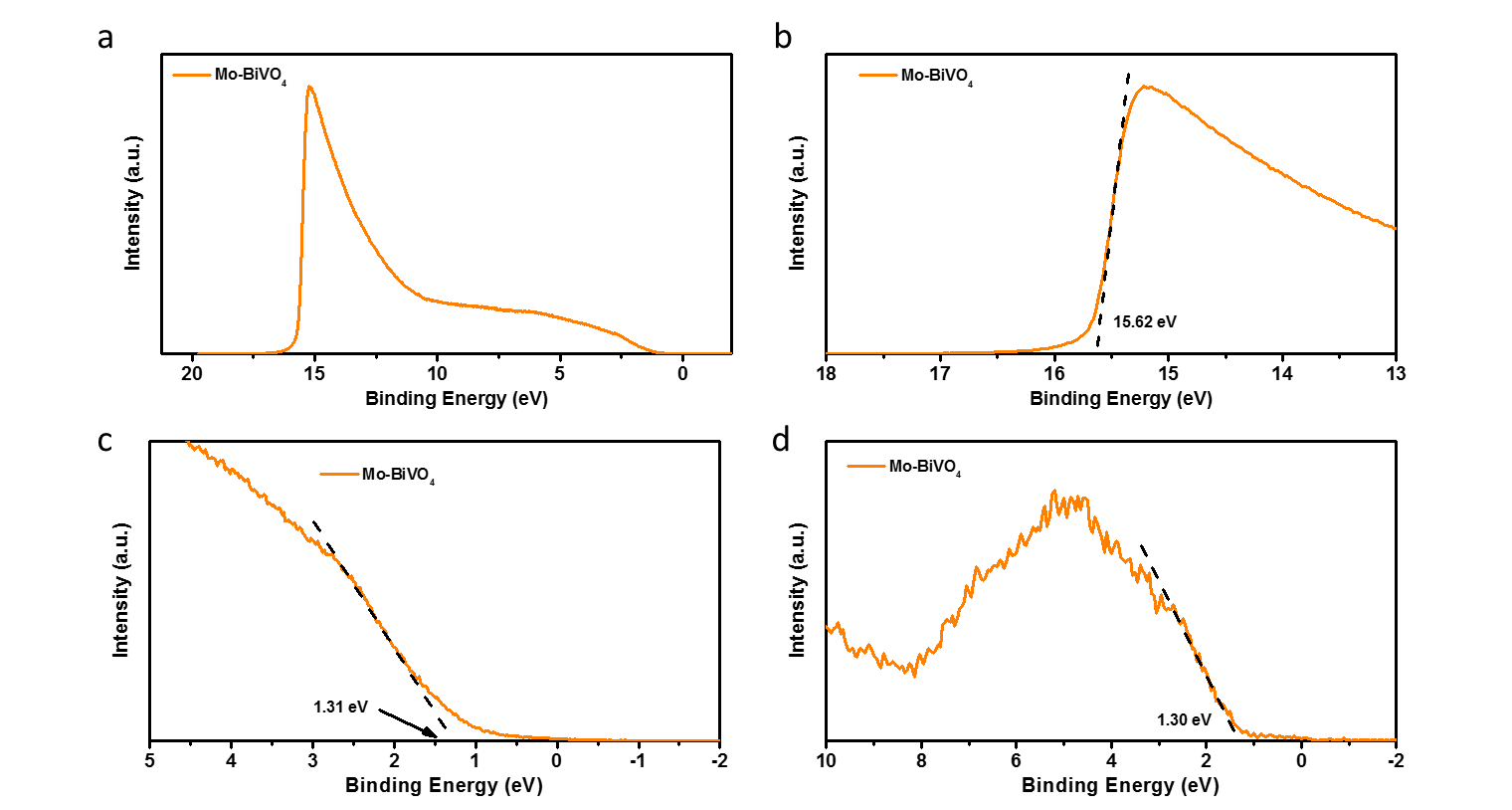


**Supplementary Figure 11.** UPS and XPS valence band spectra. **a-c** UPS and **d** XPS valence band spectra of Mo-BiVO_4_.


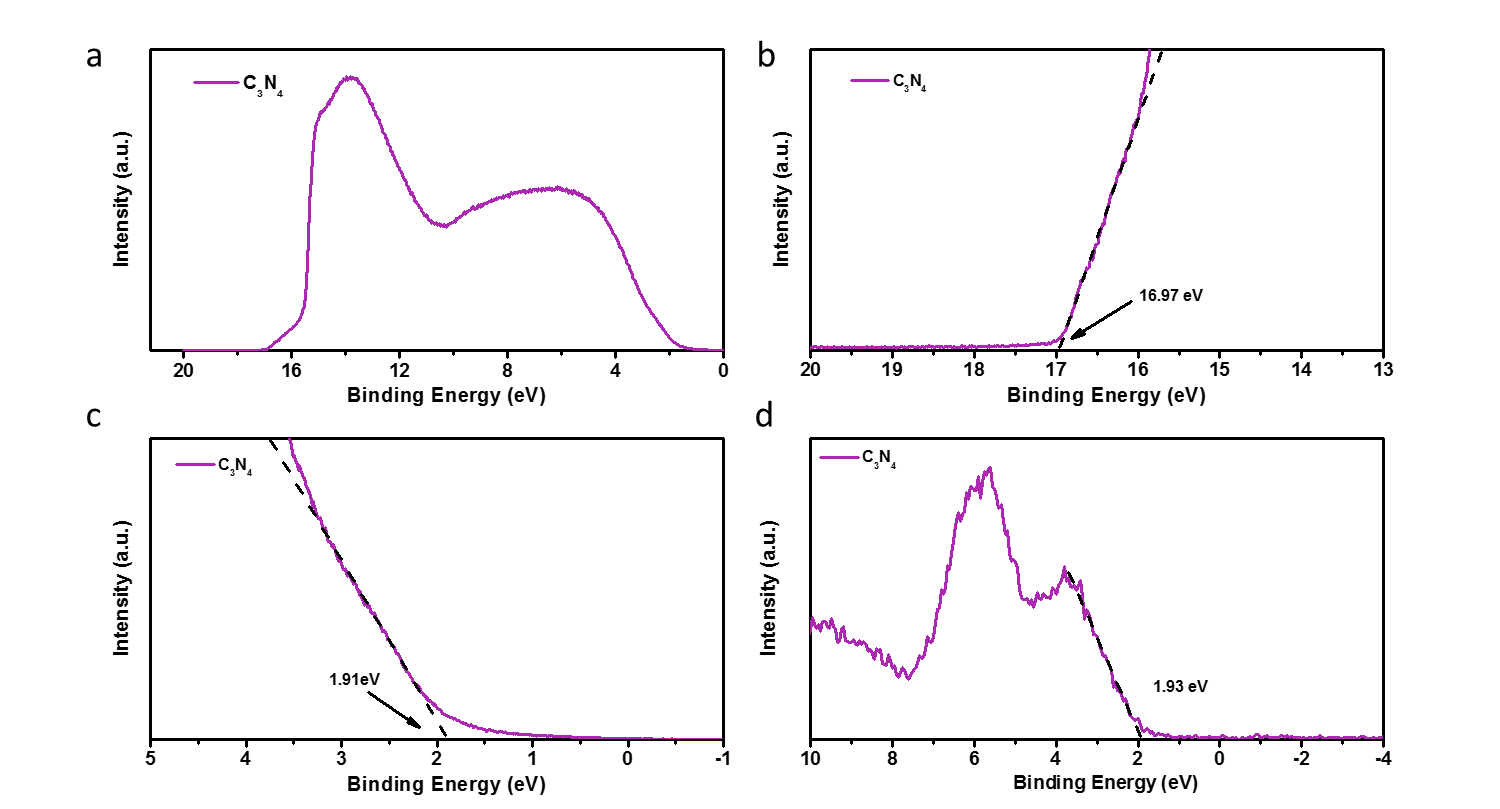


**Supplementary Figure 12.** UPS and XPS valence band spectra. **a-c** UPS and **d** XPS valence band spectra of C_3_N_4_.


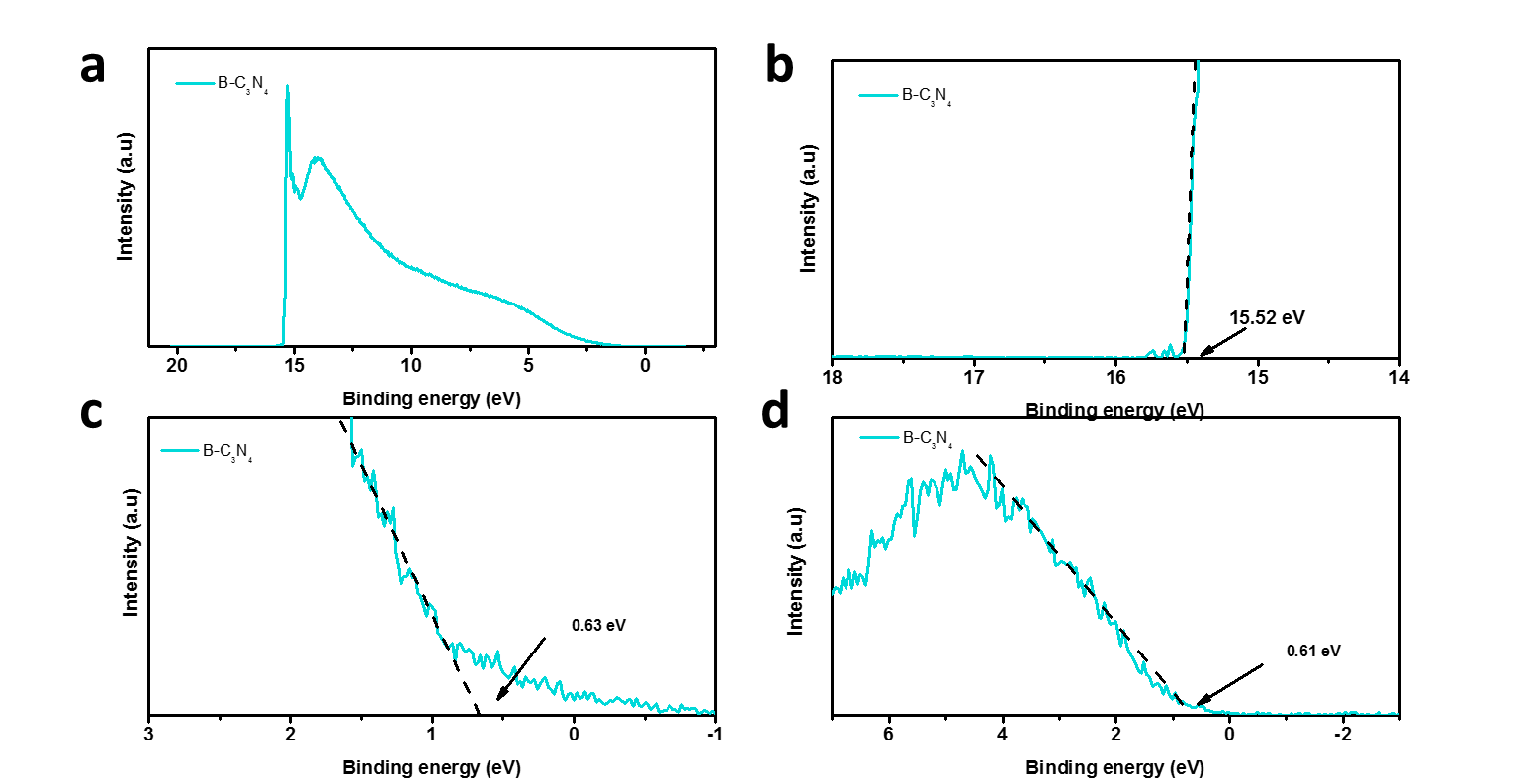


**Supplementary Figure 13.** UPS and XPS valence band spectra. **a-c** UPS and **d** XPS valence band spectra of B-C_3_N_4_.


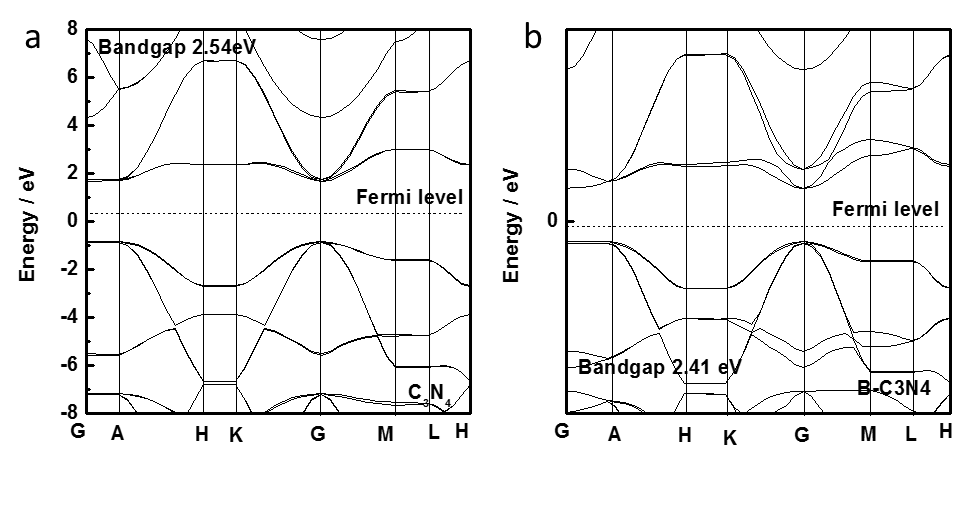


**Supplementary Figure 14.** Band structures. Band structures of **a** C_3_N_4_ and **b** B-C_3_N_4_.


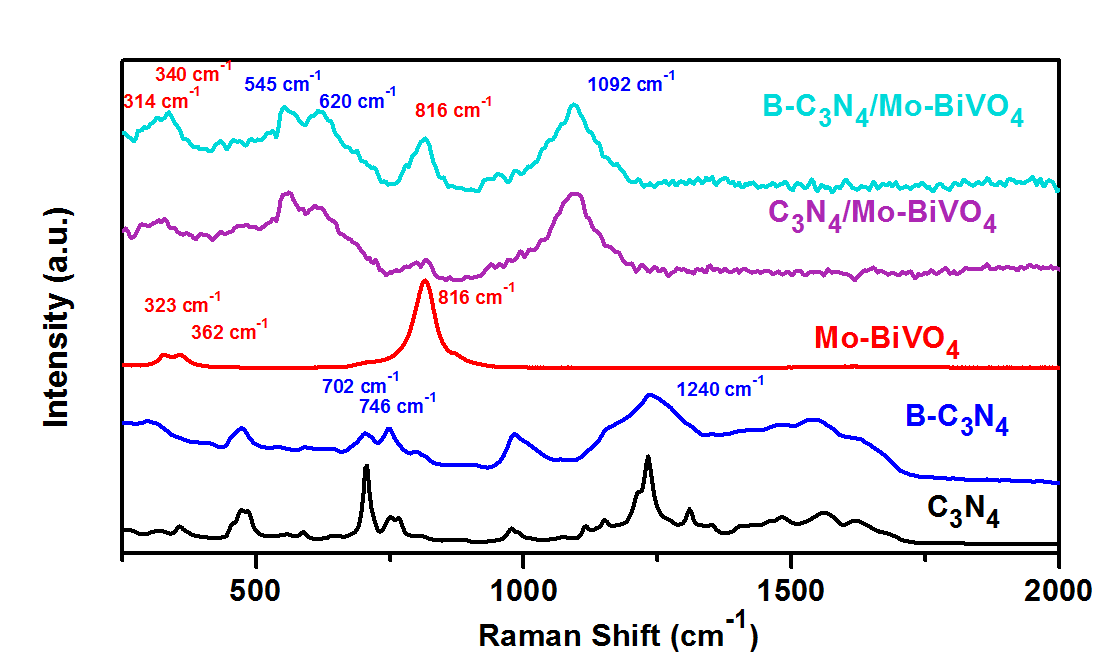


**Supplementary Figure 15.** Raman-scattering spectra of the samples. Enlarged Raman-scattering spectra of Mo-BiVO_4_, C_3_N_4_, B-C_3_N_4_, C_3_N_4_/Mo-BiVO_4_ and B-C_3_N_4_/Mo-BiVO_4_ in the range of 50-2000 cm^-1^.


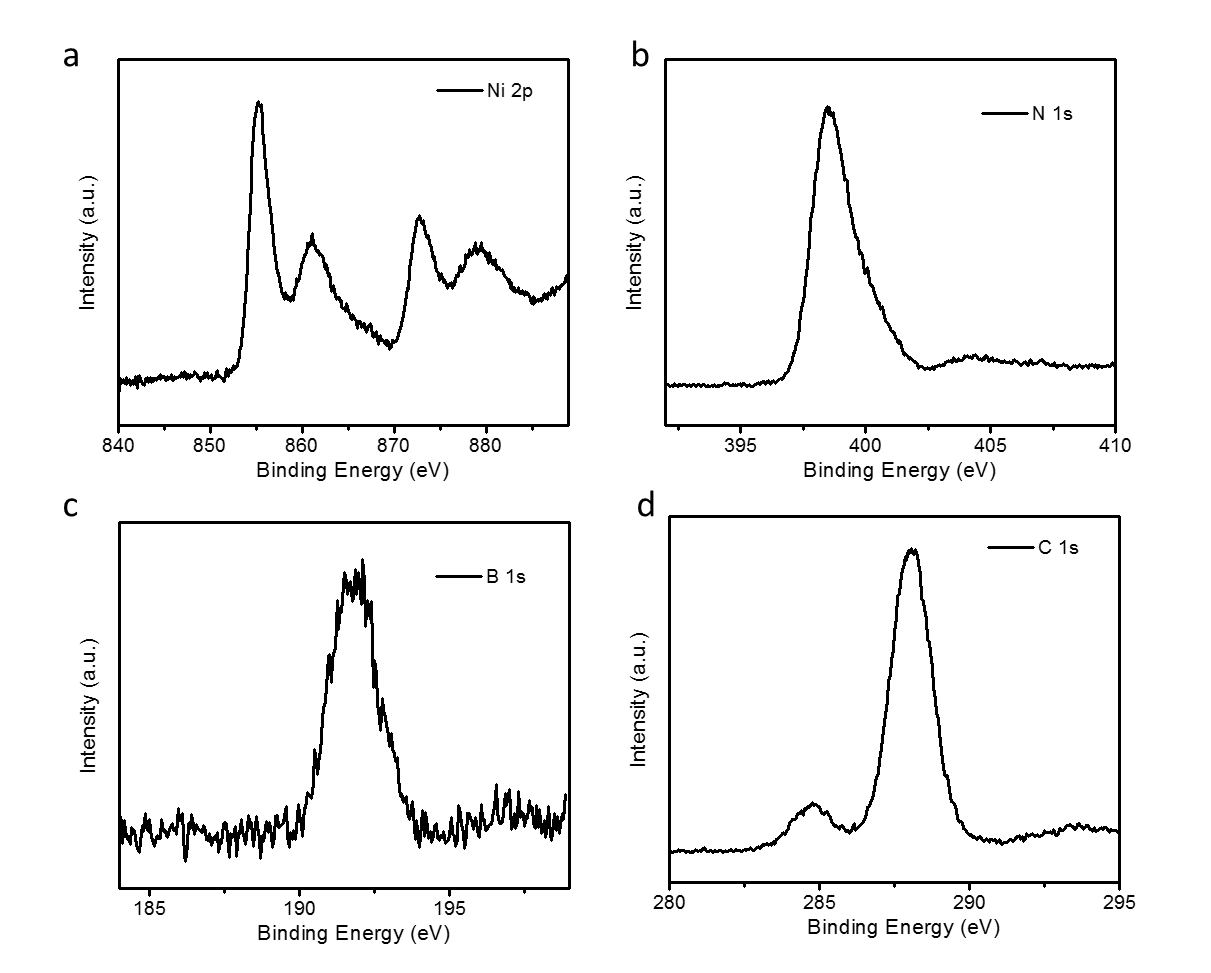


**Supplementary Figure 16.** High-resolution XPS spectra of NiFeO_x_/B-C_3_N_4_/Mo-BiVO_4_ photoanode. High-resolution XPS spectra of **a** Ni 2p, **b** N 1s, **c** B 1s and **d** C1s.


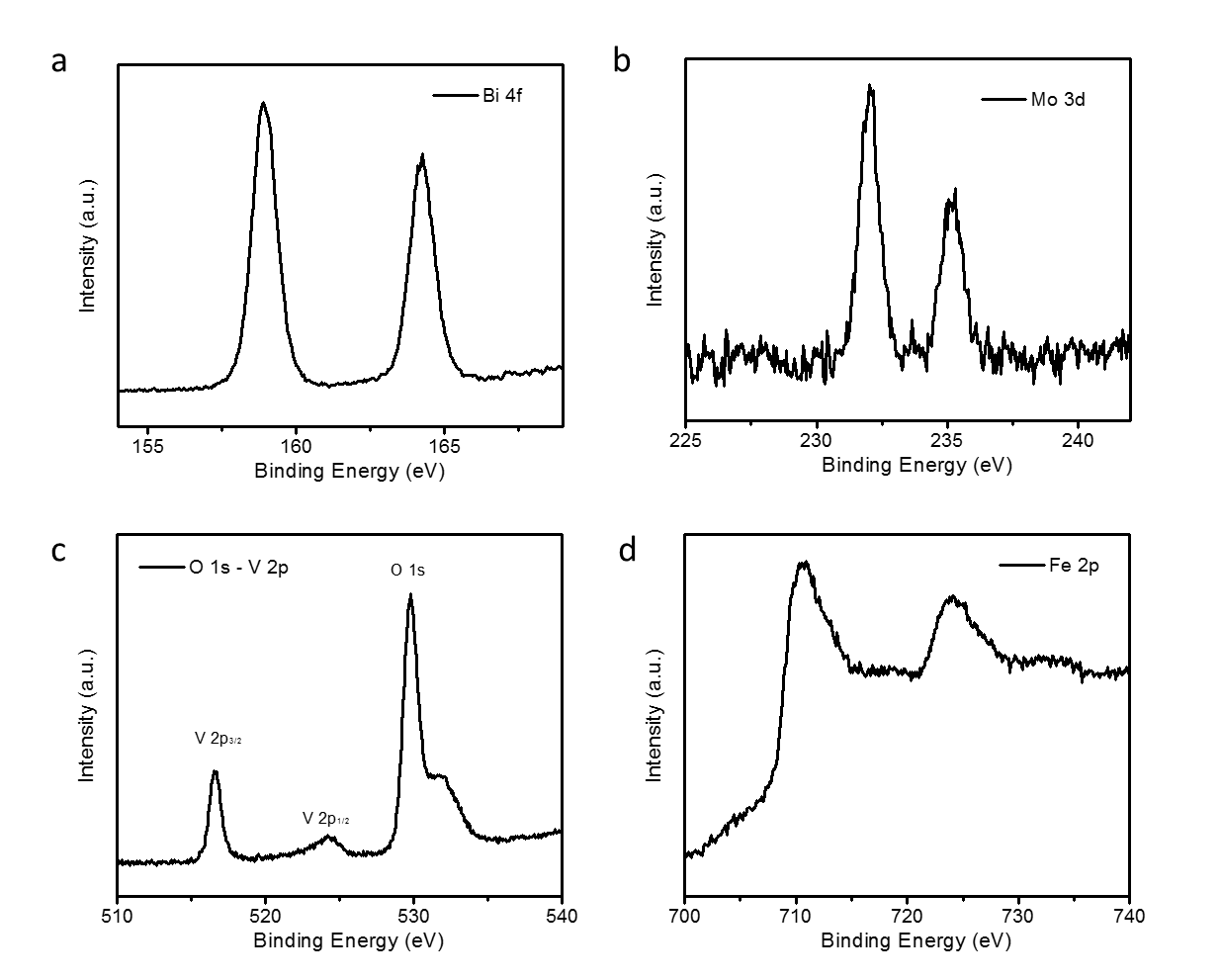


**Supplementary Figure 17.** High-resolution XPS spectra of NiFeO_x_/B-C_3_N_4_/Mo-BiVO_4_ photoanode. High-resolution XPS spectra of **a** Bi 4f, **b** Mo 3d, **c** O 1s - V 2p and **d** Fe 2p.


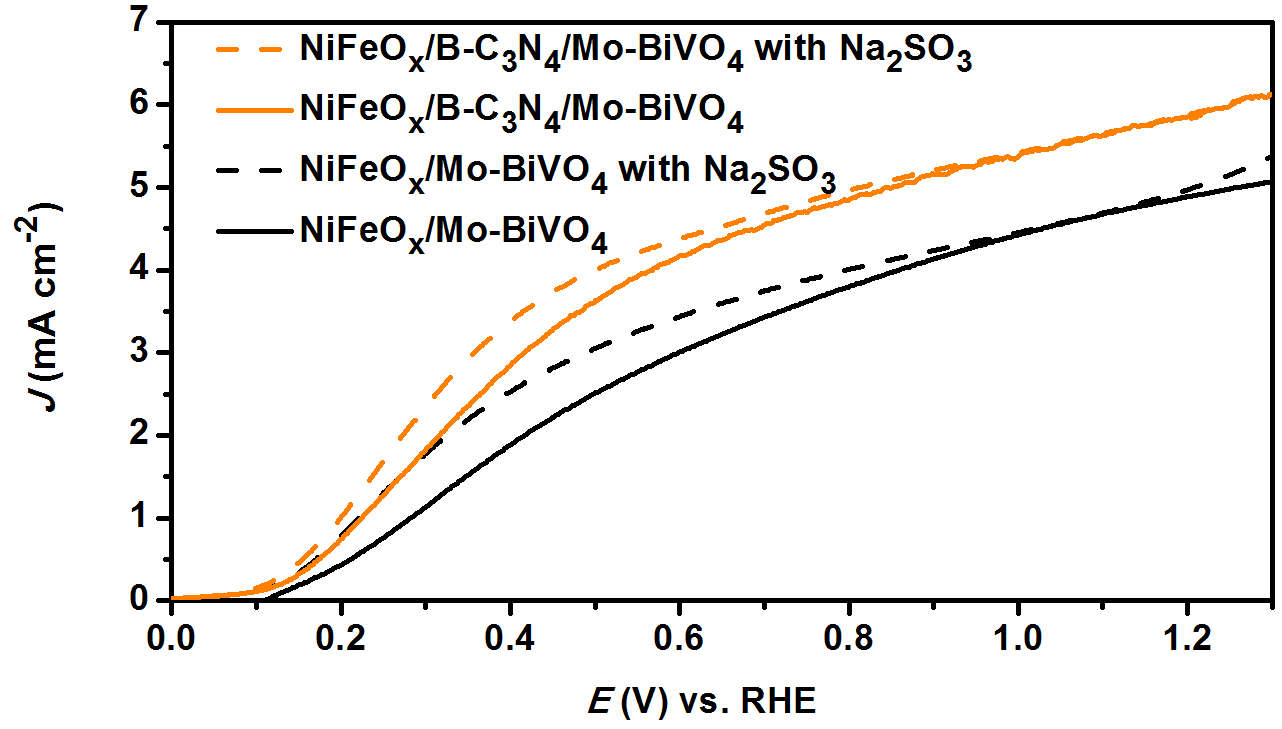


**Supplementary Figure 18.** LSV curves of the samples with and without hole scavenger. LSV curves of the NiFeO_x_/Mo-BiVO_4_ and NiFeO_x_/B-C_3_N_4_/Mo-BiVO_4_ recorded at a scan rate of 25 mV s^-1^ in PPB solution without (solid line) and with (dash line) Na_2_SO_3_ as a hole scavenger (pH = 7), which shows that the B-C_3_N_4_ can not only increase the photocurrent density in the low biased voltage range (0.1 ~ 0.8 V *vs.* RHE) by increasing the separation efficiency of photoanode, but also increase the photocurrent density in the high biased voltage range (0.8 ~ 1.3 V *vs.* RHE) by increasing the light absorption of photoanode.


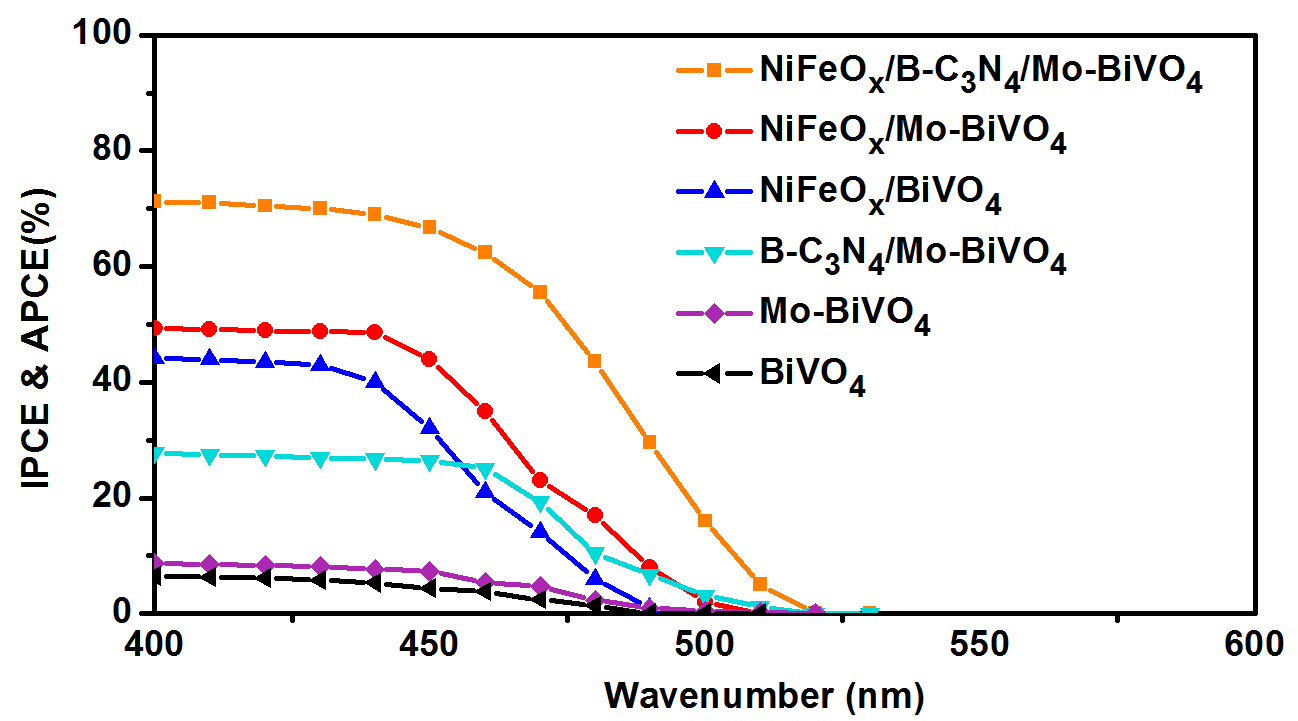


**Supplementary Figure 19.** IPCEs of the BiVO_4_-based photoanodes. IPCEs of NiFeO_x_/B-C_3_N_4_/Mo-BiVO_4_, NiFeO_x_/Mo-BiVO_4_, NiFeO_x_/ BiVO_4_, B-C_3_N_4_/Mo-BiVO_4_, Mo-BiVO_4_ and BiVO_4_ at 0.54 V *vs.* RHE in PPB solution (pH = 7), which shows that the B-C_3_N_4_ can increase the charge separation efficiency and light absorption of photoanode. On the other hand, the NiFeO_x_ cannot increase light absorption of photoanode, and it only plays the role of a co-catalyst (OEC) here.


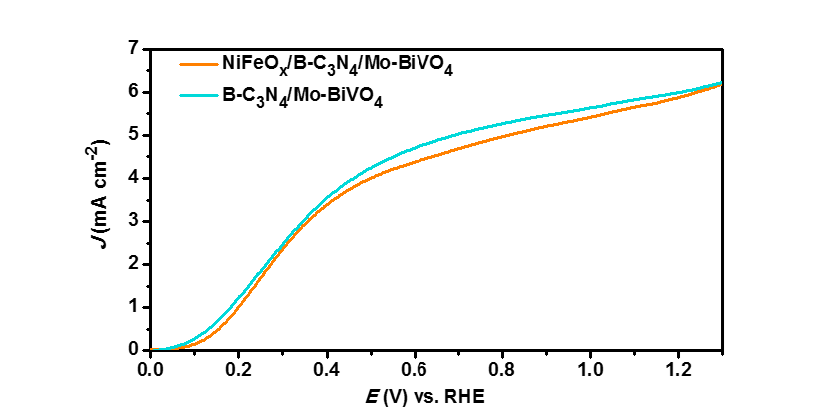


**Supplementary Figure 20.** LSV curves of the samples with hole scavenger. LSV curves of the B-C_3_N_4_/Mo-BiVO_4_ and NiFeO_x_/B-C_3_N_4_/Mo-BiVO_4_ recorded at a scan rate of 25 mV s^-1^ in PPB solution with Na_2_SO_3_ as a hole scavenger (pH = 7), which shows that the photocurrent density of samples measured in solution with Na_2_SO_3_ will decline after the NiFeO_x_ layer deposition.

**Supplementary Figure 21.** Deviation of photocurrent of NiFeO_x_/B-C_3_N_4_/Mo-BiVO_4_. The deviation of photocurrent of NiFeO_x_/B-C_3_N_4_/Mo-BiVO_4_ photoanode by taking the standard values of measurements on four different samples.

**Supplementary Figure 22.** Original UV-vis spectra. The original UV-vis spectra of BiVO_4_ and Mo-BiVO_4_.


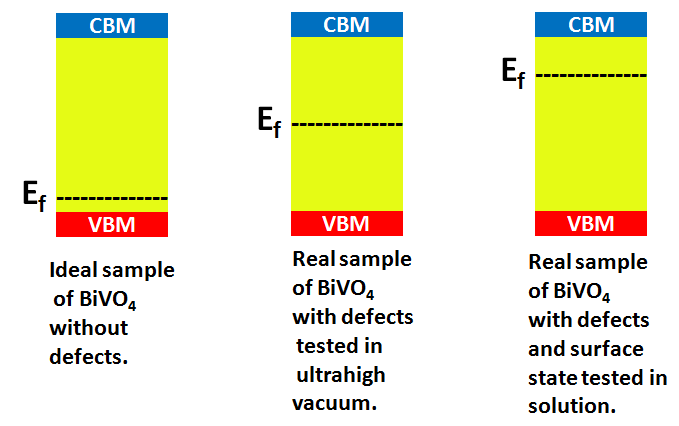


**Supplementary Figure 23.** Band structures of ideal and real BiVO_4_ samples. Band structures of ideal BiVO_4_ sample without defects (left), real BiVO_4_ sample with defect exposed to ultrahigh vacuum (middle) and to solution (right).


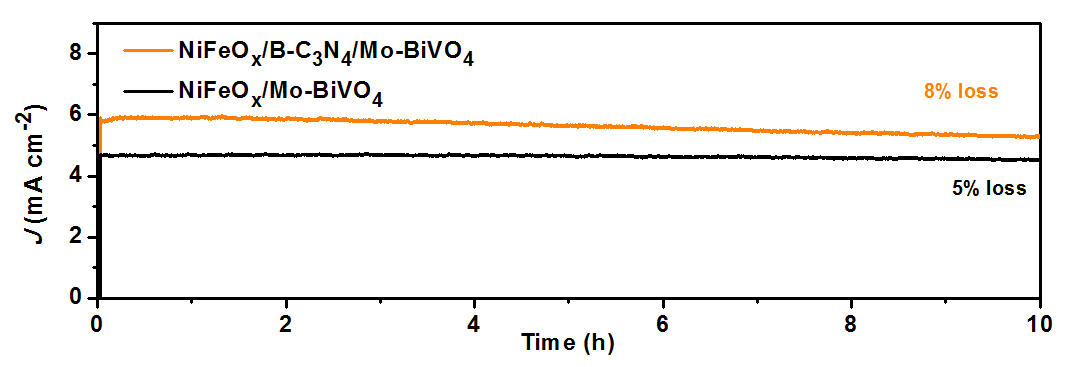


**Supplementary Figure 24.** Chronoamperometry (i-t) curves of the samples in PPB solution. Chronoamperometry (i-t) curves of NiFeO_x_/B-C_3_N_4_/Mo-BiVO_4_ (orange line) and NiFeO_x_/Mo-BiVO_4_ (black) photoanodes collected at 1.23 V *vs.* RHE under AM 1.5G illumination in PPB solution (pH = 7).

**Supplementary Figure 25.** LSV curve in PPB solution. LSV curve of two electrodes (NiFeO_x_/B-C_3_N_4_/Mo-BiVO_4_ photoanode and Pt cathode) configuration in PPB solution (pH 7).

**Supplementary Figure 26.** ABPE measurement. ABPE measurement of two electrodes (NiFeO_x_/B-C_3_N_4_/Mo-BiVO_4_ photoanode and Pt cathode) configuration in PPB solution (pH 7).

**Supplementary Figure 27.** Electrochemical Impedance spectra. Electrochemical Impedance spectra of BiVO_4_, 0.05% Mo-BiVO_4_, 0.1% Mo-BiVO_4_ and 0.5% Mo-BiVO_4_ in PPB solution with Na_2_SO_3_ hole scavenger under illuminated condition at 0.6 vs. RHE.

**Supplementary Figure 28.** Mott-Schottky plots. Mott-Schottky plots for pure BiVO_4_, 0.05% Mo-BiVO_4_, 0.1% Mo-BiVO_4_ and 0.5% Mo-BiVO_4_.


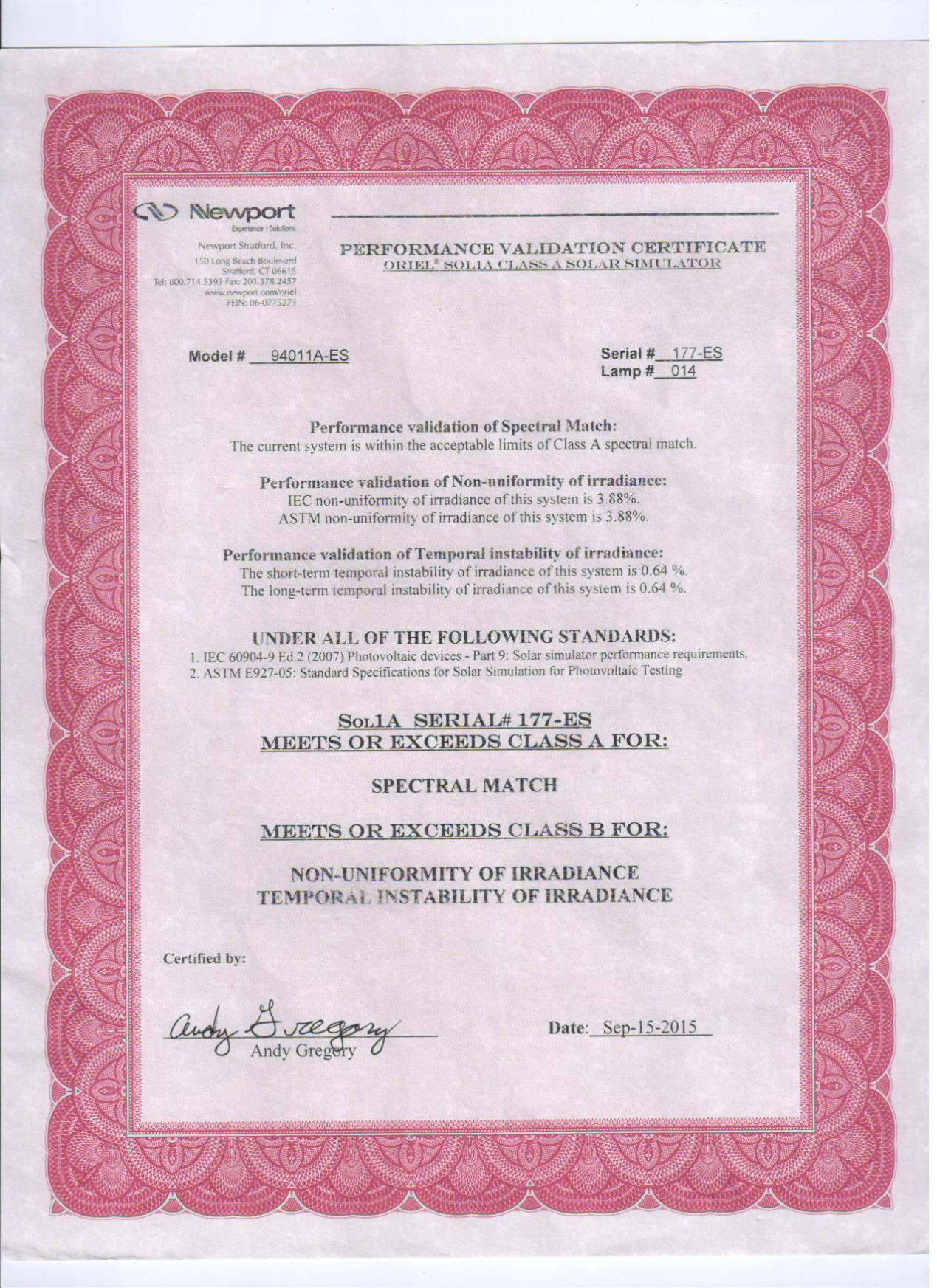


**Supplementary Figure 29.** Performance validation certificate. Performance validation certificate of AM 1.5G solar simulator (Newport, LCS 100 94011A).

**Supplementary Table 1.** ICP-AES results of Mo and Bi elements.

| Sample name |  | Analytical result of Mo (mg L^-1^) | Analytical result of Bi (100 mg L^-1^) | Mo/Bi concentration  (atomic ratio) | Mo/Bi concentration  Average |
| --- | --- | --- | --- | --- | --- |
| 0.05% Mo-BiVO_4_ | Test 1 | 0.202 | 4.09 | 0.108% | 0.110% |
|  | Test 2 | 0.206 | 3.96 | 0.113% |  |
| 0.1% Mo-BiVO_4_ | Test 1 | 0.273 | 4.03 | 0.148% | 0.156% |
|  | Test 2 | 0.299 | 3.94 | 0.165% |  |
| 0.5% Mo-BiVO_4_ | Test 1 | 1.16 | 4.29 | 0.589% | 0.604% |
|  | Test 2 | 1.12 | 3.94 | 0.619% |  |

**Supplementary Video 1.** The water splitting device in potassium phosphate solution without hole scavenger (pH = 7) (RT) at 0.6V *vs.* RHE under AM 1.5G irradiation (100 mW cm^-2^).

**Supplementary References**

1. Kim, T. W. & Choi, K-S. Nanoporous BiVO_4_ photoanodes with dual-layer oxygen evolution catalysts for solar water splitting. *Science* **343**, 990-994 (2014).

2. Ye, K-H. et al*.* Carbon quantum dots as a visible light sensitizer to significantly increase the solar water splitting performance of bismuth vanadate photoanodes. *Energy Environ. Sci.***10**, 772-779 (2017).

3. Standard, A. *Annual book of ASTM standards* **2008**, 12.

4. Shi, X. et al. Understanding the synergistic effect of WO 3–BiVO 4 heterostructures by impedance spectroscopy. *Phys. Chem. Chem. Phys.***18**, 9255-9261 (2016).

5. Zhong, D. K., Choi, S. & Gamelin, D. R. Near-complete suppression of surface recombination in solar photoelectrolysis by “Co-Pi” catalyst-modified W: BiVO_4_. *J. Am. Chem. Soc.***133**, 18370-18377 (2011).

6. Kuang, Y. et al. A Front-Illuminated Nanostructured Transparent BiVO_4_ Photoanode for > 2% Efficient Water Splitting. *Adv. Energy Mater.***6**, 1501645 (2016).

7. Kresse, G. & Furthmuller, J. *VASP the Guide* Vienna University of Technology: Vienna, 2001.

8. Ping, Y., Rocca, D., & Galli, G. Electronic excitations in light absorbers for photoelectrochemical energy conversion: first principles calculations based on many body perturbation theory. *Chem. Soc. Rev.***42**, 2437-2469 (2013).

9. Pham, T. A., Ping, Y., & Galli, G. Modelling heterogeneous interfaces for solar water splitting. *Nat. Mater.***16**, 401-408 (2017).

10. Grimme, S. Semiempirical GGA-type density functional constructed with a long-range dispersion correction. *J.Comput.Chem.***27**, 1787-1799 (2006).

11. Park, H. S. et al. Factors in the metal doping of BiVO_4_ for improved photoelectrocatalytic activity as studied by scanning electrochemical microscopy and first-principles density-functional calculation. *J. Phys. Chem. C***115**, 17870-17879 (2011).
